# Multi-Label Classification of Adverse Drug Reactions Using a Hybrid Approach Integrating Support Vector Machine, Random Forest and Extreme Gradient Boosting

**DOI:** 10.3390/ph19101566

**Published:** 2026-10-01

**Authors:** Ana-Maria Cucos, Zsolt Gáll, László Barna Iantovics

**Affiliations:** 1 Doctoral School of Letters, Humanities and Applied Sciences, George Emil Palade University of Medicine, Pharmacy, Sciences and Technology of Targu Mures, 540142 Targu Mures, Romania; ana_mariacucos@yahoo.com; 2 Department of Pharmacology and Clinical Pharmacy, Faculty of Pharmacy, George Emil Palade University of Medicine, Pharmacy, Sciences and Technology of Targu Mures, 540142 Targu Mures, Romania; 3 Electrical Engineering and Information Technology Department, George Emil Palade University of Medicine, Pharmacy, Sciences and Technology of Targu Mures, 540142 Targu Mures, Romania; barna.iantovics@umfst.ro

**Keywords:** adverse drug reaction prediction, random forest, support vector machine, molecular fingerprints and descriptors, ensemble learning

## Abstract

**Background/Objectives**: Predicting adverse drug reactions (ADRs) is essential for drug safety, but challenging due to the rarity and diversity of certain clinical manifestations. This study aims to enhance multi-label ADR detection by introducing and evaluating biologically inspired strategies, specifically Balanced Thresholds (BT) and Weighted Predictions (WP), integrated into a hybrid ensemble model. **Methods**: We proposed and developed a hybrid architecture combining Random Forest (RF), Extreme Gradient Boosting (XGB), and Support Vector Machine (SVM). To handle rare ADRs without data leakage, balanced thresholds and weighting parameters were calculated exclusively on training folds within a repeated 5 × 5-fold cross-validation framework. Statistical validation was performed using Friedman and Nemenyi tests. The main motivation for this hybrid architecture was to emphasize model diversity, complementary learning mechanisms, error reduction, and generalization. **Results**: The experimental results demonstrate that the adjusted strategies significantly increase recall compared to baseline models, particularly for rare ADRs. Specifically, the Weighted Predictions strategy achieved near-perfect recall (up to 0.99) for low-prevalence disorders like RSBD and ELD. While the baseline model maintained higher overall precision, the adjustments narrowed the performance gap between frequent and rare reactions, doubling the recall for the most challenging categories. Friedman tests confirmed significant differences in model sensitivity. **Conclusions**: Integrating biological knowledge into hybrid architectures provides a framework that allows practitioners to prioritize sensitivity in high-risk scenarios and improve the detection of clinically relevant, rare ADRs.

## 1. Introduction

Adverse drug reactions (ADRs) are defined as being harmful and unintended responses to a medication that occur at doses normally used for treatment, diagnosis, or prevention of disease. ADRs can range from mild side effects, such as nausea or headache, to severe, life-threatening reactions, such as organ failure [1,2,3].

Multiple challenges are associated with ADRs. Detection and reporting remain difficult, as many ADRs are underreported, especially mild or delayed reactions. Predictability is also limited, because some ADRs are unpredictable and may occur even in patients without known risk factors. Polypharmacy further increases the risk of interactions and ADRs, since patients often use multiple medications simultaneously. In addition, patient variability, such as genetic, age-related, or disease-related factors, can influence susceptibility to ADRs. Finally, monitoring and managing ADRs require systematic pharmacovigilance, which can be resource-intensive [4].

Predicting ADRs early is important not only for patient safety, but also for guiding the design of safer drugs. In recent years, machine learning (ML) has become a popular and valuable tool in the context of ADR prediction, helping researchers discover patterns between molecular structures and their potential side effects. More broadly, ML and deep learning approaches have shown increasing utility in the analysis of complex biological data, including genomic variant detection and annotation [5]. Early structure-based methods demonstrated that chemical fragments and molecular fingerprints can support large-scale side-effect prediction from drug structure. However, a recent study found that fingerprint models are more useful for ranking drugs for an individual side effect than for accurately identifying all present and absent effects across a broad label space [6].

In our previous studies [7,8], we analyzed different ML models for ADR prediction. In our 2024 research [7], we focused on Random Forest (RF), Gradient Boosted Trees, and neural networks such as Feedforward Neural Networks and Convolutional Neural Networks. In a follow-up study in 2025 [8], we explored Support Vector Machine (SVM), Adaptive Boosting, and Logistic Regression. Our studies concluded that each model captured different patterns in the data, essentially exploring the molecular information from different perspectives. RF proved to be particularly resilient when dealing with noisy or redundant descriptors, as its ensemble nature allowed it to filter out irrelevant data that might otherwise skew results. Meanwhile, SVM excelled at identifying subtle, non-linear trends within high-dimensional spaces, picking up on complex chemical nuances that simpler models might have missed. The fact that no single model was clearly the best in all situations highlights the inherent complexity of drug-molecule interactions. This diversity in performance suggested that relying on a single algorithm might be limiting. Combining these models in a hybrid ensemble framework may therefore provide more reliable and comprehensive predictions [9,10].

This research introduces a baseline hybrid ensemble that combines RF, Extreme Gradient Boosting (XGB), and SVM models. In addition to this baseline, we implemented adaptations such as label-specific thresholding and weighting. These adjustments were biologically inspired in the sense that they reflect the natural variance in how ADRs manifest in the human population. In a clinical setting, side effects are not distributed uniformly; some are rare but severe, while others are common and mild. By using label-specific thresholding, we mirrored the biological reality that different physiological systems have different trigger points for a reaction. Similarly, weighting allowed us to account for label prevalence, ensuring that rarer, often more critical biological signals were not drowned out by more frequent, less significant ones. This approach helped the models move beyond simple pattern matching and better align with the actual frequency and impact of drug responses in living organisms.

The main contributions of this study are: (i) a hybrid RF–XGB–SVM ensemble for multi-label ADR prediction; (ii) biologically motivated, label-aware thresholding and weighting to address class imbalance; (iii) recall-oriented optimization for high-risk pharmacovigilance scenarios; and (iv) statistical validation across folds using Friedman and Nemenyi tests.

This study also highlights that even well-optimized models can complement each other when combined thoughtfully. Hybrid modelling, paired with careful statistical evaluation, can provide more reliable predictions while remaining straightforward to implement.

## 2. Results

### 2.1. Predictive Performance on the Side Effect Resource Dataset

In this section, we present the results for the independent RF, SVM, and XGB models, the baseline hybrid model, and the biologically inspired adjustments.

Table 1 presents the mean performance including the standard deviation (SD) of each the standalone RF, SVM and XGB models across the 25 repeated cross-validation iterations.

Across the 25 iterations, performance varies significantly depending on the specific system organ class (SOC) and ML algorithm (Table S1). For eye disorders (ED) and blood and lymphatic system disorders (BLSD), models like RF and XGB achieve stable performance, with high recall and solid F1-scores and area under the precision–recall curve (AUC-PR) values, showing they can effectively catch ADRs. Reproductive system and breast disorders (RSBD) also shows reliable scores across algorithms like RF and XGB. On the other hand, ear and labyrinth disorders (ELD) and ED struggle heavily, especially when using SVM, where recall drops close to 0 in several iterations, highlighting the difficulty these models face with class imbalance.

Table 1 also highlights a clear performance issue for the SVM model when predicting the ELD class, characterized by a precision of 1.00 and a recall close to 0. This happens primarily because ELD is a low-prevalence SOC in the Side Effect Resource dataset (SIDER) (~47% prevalence). When standard SVMs deal with imbalanced data, the decision boundary naturally shifts to minimize overall errors, prioritizing the majority class. As a result, the model becomes overly cautious, labeling almost every compound as negative and only assigning positive predictions to a small number of cases where confidence is absolute.

Table S2 reports the iteration-level performance of the baseline hybrid model across 25 repeated cross-validation iterations. The model showed stable performance across these iterations. The BLSD and ED SOCs achieve the highest recall values, exceeding 0.90, which indicates a strong ability of the model to correctly detect these classes. Precision remains at a relatively constant level across all categories, with averages ranging between 0.64 and 0.68. F1-score and AUC-PR show solid results for BLSD, ED, and RSBD, with values generally exceeding 0.70. The only notable exception is the ELD label, which presents a lower recall of approximately 0.45, suggesting that this class was more difficult for the model to recognize correctly, consistent with its lower prevalence and greater prediction difficulty (Figure 1).

Based on the results presented in Table S3, the balanced thresholds strategy led to a substantial shift in the classification dynamics, focusing on maximizing the detection capability across all ADR categories. As illustrated in Figure 2, this adjustment successfully drove the average recall values to near-optimal levels, exceeding 0.94 for RSBD, ELD, and approaching perfection for BLSD and ED. The model drastically reduced false negatives, which is a critical requirement in biomedical screening where missing an active toxic endpoint carries high clinical risk.

However, this high sensitivity came with a trade-off in precision, which had a proportional decrease, ranging between 0.48 (for ELD) and 0.62 (for BLSD), due to an increase in false positive classifications. The overall F1-score remained stable, between 0.61 and 0.77, demonstrating a well-calibrated compromise between sensitivity and specificity under the adjusted decision thresholds. The global AUC-PR metrics remained consistent with previous evaluations, confirming that the intrinsic ranking capability of the underlying ensemble architecture is preserved regardless of operational threshold tuning.

As shown in Table S4, the weighted predictions strategy across the 25 iterations shows consistent performance across the four SOCs. Recall remains high, reaching nearly 1.0 for BLSD, ED, and ELD, and approximately 0.98 for RSBD, demonstrating the model’s strong capability to capture positive instances. Precision stays at a more conservative level, particularly for minority classes such as ELD, where, based on Figure 3, averages hover around 0.45, reflecting a higher rate of false positives. The F1-score maintains a reasonable balance between precision and sensitivity, between 0.70 and 0.78, while AUC-PR values highlight that the model preserves solid ranking performance for major classes despite the weighting adjustments.

### 2.2. Statistical Tests for Model Comparison

#### 2.2.1. Hybrid Ensemble Versus Individual Models

First, after independently evaluating the individual models and the baseline hybrid model, we performed a rigorous statistical significance analysis using a repeated k-fold cross-validation framework (5 × 5-fold, yielding *n* = 25 independent performance data points per metric), following recent methodological recommendations for robust predictive modeling in computational biology. To compare the model distributions, we employed the non-parametric Wilcoxon Signed-Rank Test as a robust alternative to the paired Student’s *t*-test, bypassing the strict normality assumptions required by parametric methods for small sample sizes. Furthermore, the Wilcoxon test is ideally suited for dependent observations, as the performance scores of the compared models were evaluated across identical cross-validation splits. The test evaluates whether the median difference between the paired sets of observations deviates significantly from zero. For each label and evaluation metric (Recall, Precision, F1-score, and AUC-PR), the baseline hybrid model was compared against each standalone model (RF, SVM, and XGB). The null hypothesis assumed no significant difference between the median performances of the compared models, whereas the alternative hypothesis assumed a statistically significant difference. A standard threshold of *p* < 0.05 was adopted to determine statistical significance, with the expanded sample size (*n* = 25) ensuring adequate statistical power to detect meaningful performance variations. Table 2 presents the results of the paired Wilcoxon test:

Table 2 details the results of the Wilcoxon signed-rank tests conducted on the repeated 5 × 5 cross-validation data, comparing the performance of the hybrid model against the standalone RF, SVM, and XGB algorithms. Across the evaluated ADR categories, the hybrid model shows a clear and consistent advantage over SVM. For SVM, nearly all comparisons yield statistically significant improvements in favor of the hybrid approach, particularly across precision, F1-score, and AUC-PR, highlighting how the hybrid architecture overcomes the limitations of standard linear or kernel-based separations in complex toxicity data. When looking at the tree-based models, RF and XGB present a more competitive challenge. In certain categories, such as ELD or specific label subsets, the standalone tree models occasionally achieve higher scores on metrics like raw recall. However, the hybrid model frequently balances this out by achieving better precision or showing superior overall performance on composite metrics. Furthermore, the inclusion of AUC-PR provides a clearer picture of performance under class imbalance, where the hybrid model maintains stable ranking capabilities. Taken together, these outcomes demonstrate that combining structural fragments with global physicochemical descriptors delivers a robust, statistically sound, and well-rounded predictive framework that performs reliably across diverse toxicological endpoints.

#### 2.2.2. Baseline Versus Balanced Thresholds Versus Weighted Predictions

To compare the performance of the three modelling strategies (Baseline hybrid model, Balanced Thresholds and Weighted Predictions) across multiple folds and metrics, we used a set of non-parametric statistical tests: the Friedman test, the Nemenyi post hoc test and the Binomial test.

We incorporated the Binomial Test to complement standard performance metrics with a statistical assessment of result reliability. Given that each prediction can be framed as a binary outcome (success/failure), the classification task can be modelled as a sequence of Bernoulli trials. This allowed us to evaluate whether the high recall values observed, approaching 0.99, could plausibly arise under a random baseline. The resulting probabilities were negligible, indicating that such performance is highly unlikely to be due to chance or favourable data partitioning. The Binomial Test serves as a validation step, supporting the conclusion that the improvements achieved by the Balanced Thresholds and Weighted Predictions strategies indicate an increase in the model’s sensitivity.

First, we used the Friedman test [11] as a global statistical test to determine whether there were overall performance differences among the models for each disorder category and each evaluation metric. The Friedman test is particularly appropriate in this context because all models were evaluated on the same cross-validation splits, resulting in paired and dependent observations. When the Friedman test indicated statistically significant differences (*p* < 0.05) we applied a Nemenyi post hoc test [12] to identify which specific model pairs differed significantly. In addition, a binomial test was applied to assess whether one model consistently outperformed another across folds. Table 3 shows the comparative performance of the models.

Table 3 demonstrates that applying Balanced Thresholds and Weighted Predictions increases recall across all disorder categories, reaching up to 1.00, as confirmed by highly significant Friedman and binomial tests (*p* < 0.0001). However, this substantial gain in sensitivity comes with a clear trade-off, leading to a noticeable decrease in precision. Meanwhile, the F1-score and AUC-PR remain stable or highly competitive relative to the Baseline Hybrid Model, indicating that these optimization strategies successfully boost the model’s detection rate for critical toxicological effects without collapsing overall performance.

The Friedman–Nemenyi ranking analyses are summarized visually in Supplementary Figures S1–S4. These Critical Difference diagrams show the average ranks of the Baseline Hybrid model, Balanced Thresholds strategy, and Weighted Predictions strategy for Recall, Precision, F1-score, and AUC-PR, respectively. Consistent with Table 3, the diagrams highlight the recall advantage of Weighted Predictions and Balanced Thresholds, the higher precision of the Baseline Hybrid model, and the comparatively small rank differences among the strategies for F1-score and AUC-PR.

### 2.3. Model Interpretability

To understand the logic behind the ensemble’s predictions, we evaluated the post hoc feature importance scores from the underlying tree-based estimators (RF and XGB) within the multi-label wrapper. This analysis allows us to assess whether the model relies on plausible chemical rules rather than arbitrary statistical noise when flagging ADRs across the SOCs. As shown in Figure 4, Figure 5, Figure 6, Figure 7, Figure 8, Figure 9, Figure 10 and Figure 11, the ensemble automatically adjusts its feature selection pattern based on the distinct biological and chemical drivers of each target toxicity category:

Reproductive system and breast disorders (Figure 4): The predictions for this endocrine-sensitive category are primarily based on Layered_Bit_119, alongside a balanced mix of RDKit structural bits and global lipophilicity (MolLogP). This combination points toward a sensitivity to specific localized molecular shapes and steroid-mimetic patterns that interact with hormone receptors, supported by global lipophilic properties.

As shown in Figure 5, for RSBD, looking closer at the representative molecular structures, it is revealed that the model is locking onto complex polycyclic and steroid-like backbones (Layered_Bit_119), which mimic natural reproductive and sex hormones, facilitating deep binding into hormone receptor pockets. The secondary structural features point toward nitrogen-rich heterocyclic systems featuring carboxamide groups (RDKit_Bit_612), as well as flexible substituted benzylamine and carbamate linkers (RDKit_Bit_162) that provide essential hydrogen-bonding networks. The prominence of chlorinated aromatic motifs (RDKit_Bit_540) highlights the model’s sensitivity to halogenated hydrophobic fragments often associated with endocrine-disrupting potentials [13,14,15].

Ear and labyrinth disorders (Figure 6): Inner-ear toxicities show a distributed feature pattern led by MolLogP, followed by topological descriptors such as Avalon_Bit_1445 and polar surface area (TPSA). This underlines a clear pharmacokinetic mechanism where toxicity depends heavily on the drug’s physical capacity to cross the blood–labyrinth barrier.

As shown in Figure 7, for ear and labyrinth disorders, a deeper look at the extracted features reveals that the machine learning model focuses heavily on global pharmacokinetic drivers and specific electrostatic interactions. Specifically, global physicochemical properties such as lipophilicity (MolLogP) and molecular polar surface area (TPSA) indicate that membrane permeability and polarity play key roles in mediating ototoxic profiles. Furthermore, structural fragment analysis highlights the significance of ionic species and small acidic components, such as conjugated keto-acid configurations combined with counter-ions (Avalon_Bit_1445), which alter local cellular pH and ionic transport across the endolymphatic barrier in the inner ear. At the same time, complex polycyclic scaffolds featuring heterocyclic motifs (Avalon_Bit_1827) and flexible aliphatic amine chains containing terminal alkyne or branched benzyl groups (RDKit_Bit_1419) provide the steric and hydrophobic properties necessary to interact with ion channels or sensory hair cell receptors, triggering adverse auditory manifestations such as tinnitus or sensorineural impairment [16,17,18].

Eye disorders (Figure 8): Ocular toxicities are heavily based on global physicochemical properties, with MolLogP ranking as the top feature and TPSA in second place, followed by layered and Avalon structural bits. This highlights that drug accumulation and penetration through lipid membranes and ocular tissues are primarily dictated by lipophilic transport dynamics.

In Figure 9, for ED, the interpretability analysis reveals that the model heavily weighs global pharmacokinetic descriptors such as lipophilicity (MolLogP) and topological polar surface area (TPSA). These global properties dictate the systemic distribution and penetration of drug molecules through blood–ocular barriers, such as the blood–retinal or blood–aqueous barriers. Furthermore, the structural fragment decoding highlights specific functional motifs, including ionized chloride species (Layered_Bit_1151) and polar alpha-hydroxy acid chains (Layered_Bit_1281), which can alter local ionic balance and osmotic pressure within ocular tissues. Additionally, more complex scaffolds featuring substituted diphenylmethyl piperazine derivatives with terminal carboxylic acid groups (Avalon_Bit_1748) act as crucial structural alerts. These motifs are frequently associated with off-target binding to ocular neurotransmitter receptors or accumulation in pigmented tissues (such as the uvea and retina), driving the predictive score for drug-induced ocular toxicities [19,20].

Blood and lymphatic system disorders (Figure 10): This category is led by RDKit_Bit_1768 and structural alerts like MACCS_key_117 and MACCS_Key_94. The prominent ranking of predefined MACCS keys confirms that the model successfully isolates classic chemical flags, such as reactive groups or specific heteroatom arrangement associated with blood dyscrasias and bone marrow suppression.

As shown in Figure 11, for BLSD, the interpretability analysis indicates that the model focuses on structural alerts capable of inducing hematological toxicities, immune-mediated reactions, or plasma protein binding alterations. Specifically, primary feature contributions arise from sulfonamide-containing and complex polycyclic motifs (RDKit_Bit_1768), which are well-documented structural alerts linked to drug-induced blood dyscrasias and bone marrow suppression. Furthermore, MACCS keys and RDKit structural fragments (MACCS_Key_117, MACCS_Key_94, and RDKit_Bit_1578) highlight the relevance of quaternary ammonium ions, purine-like xanthine cores, and conjugated polyene or long-chain aliphatic systems. These functional groups affect membrane stability in circulating blood cells and cellular uptake. Additionally, the presence of the global lipophilicity descriptor (MolLogP) confirms that pharmacokinetic properties govern the systemic exposure and distribution of these toxicophores within highly perfused hematopoietic and lymphoid tissues. Network-based in silico toxicology approaches further validate that capturing these multi-target structural patterns significantly enhances the predictive performance for systemic and hematological adverse drug reactions [21,22,23,24].

### 2.4. External Validation on Real-World Pharmacovigilance Data

To test the models on independent real-world data, we built an external validation dataset using the U.S. Food and Drug Administration Adverse Event Reporting System (FAERS) database, using the 2025 Q4 release [25]. FAERS is a public database containing adverse event reports, medication error reports, and quality complaints resulting in adverse outcomes. It is designed to support the FDA’s post-marketing safety surveillance program by collecting reports submitted voluntarily by healthcare professionals, patients, and manufacturers, as well as mandatory reports from pharmaceutical companies. Because it relies on spontaneous reporting, it captures real-world patient data characterized by variable reporting habits and clinical noise.

The pipeline comprised the following steps:

- Data loading and cleaning: We loaded the DRUG and REAC files and normalized the drug names by converting them to uppercase and removing extra spaces.
- Keyword-based mapping: Since the official MedDRA dictionary was not available, we used a predefined list of keywords for the 4 target System Organ Classes (SOCs) to identify adverse reactions in the text and convert them into binary labels (0 or 1).
- Pre-sampling: To avoid querying the PubChem API for tens of thousands of drugs, we extracted a sub-sample of 5000 unique drugs, maintaining a mix of positive and negative cases.
- Retrieving SMILES structures: We queried the PubChem API using the drug names to obtain their chemical structures (SMILES), adding a short delay between requests to prevent overloading the server.
- Final balanced selection: We filtered out entries without valid SMILES strings and selected a final set of 724 rows, ensuring no overlap with the SMILES strings present in the SIDER training dataset.

Figure 12 presents the number of positives vs. negatives for each label and the relative balance in percentage between positive and negative examples.

We applied the exact same models, soft-voting ensemble strategy, and optimization parameters to the external FAERS validation dataset. Table 4 summarizes the external validation performance of the baseline hybrid model, the balanced-threshold strategy, and the weighted-predictions strategy on the FAERS dataset.

Evaluating the models on the external FAERS dataset reveals a clear drop in absolute performance metrics compared to the curated SIDER benchmark, which is expected given the inherent noise, reporting biases, and spontaneous nature of real-world pharmacovigilance data. FAERS reports consist of unverified clinical suspicions, co-medications, and variable reporting standards rather than clean, expert-curated package inserts. Because of this domain shift, baseline metrics show moderate performance, particularly for categories such as RSBD and ELD, where reporting ambiguities are higher. Despite the noisy external-validation setting, the alternative strategies successfully met their primary objective by driving the recall to near-perfect levels. Across both the biological thresholding and weighted prediction strategies, the models successfully drove recall to near-perfect levels, achieving 0.97 to 1.00 for ELD and BLSD. While this increase in sensitivity naturally introduces a trade-off with precision due to false positives generated by spontaneous reporting noise, it demonstrates that the model functions as a conservative screening tool capable of capturing potential safety signals without missing critical adverse events.

## 3. Discussion

The experimental results demonstrate that integrating biologically inspired adjustments, specifically balanced thresholds and weighted predictions, significantly refines the predictive behaviour of the baseline hybrid model. While the baseline model offers a conservative and precise approach, the adjusted strategies prioritize sensitivity, which is often the primary concern in pharmacovigilance.

### 3.1. Performance Trade-Offs and Strategy Selection

Our results highlight a clear trade-off between precision and recall. The Weighted Predictions approach acts as a more aggressive strategy, making it particularly effective for detecting rare ADRs such as RSBD and ELD, which are often underrepresented and overlooked in standard models due to their low prevalence. On the other hand, the Balanced Thresholds method leads to more moderate improvements and performs better for more common conditions, such as ED and BLSD. Overall, these findings suggest that there is no universal solution. Instead, the optimal approach depends on the clinical context, particularly the prevalence of the specific disorder being targeted. This observation is consistent with recent pharmacovigilance research emphasizing that the choice and evaluation of artificial intelligence approaches for drug safety should be adapted to the specific decision context and characteristics of the target adverse event [26,27].

### 3.2. Medical and Clinical Relevance

From a medical perspective, the ability to achieve a recall of 0.99 in categories like ELD is a notable result. In clinical settings, missing a potential adverse drug reaction (a False Negative) is often far more dangerous than investigating a False Positive. The high sensitivity observed in our biologically inspired models aligns with the “precautionary principle” in medicine. By successfully identifying low-prevalence disorders, these models can act as early-warning systems, allowing clinicians to monitor at-risk patients more closely before symptoms escalate. Recent pharmacovigilance studies have similarly demonstrated the potential of machine learning and signal-detection approaches to identify adverse drug reactions that may otherwise remain difficult to detect, particularly when analyzing large-scale spontaneous reporting databases [27]. Furthermore, the stability of the F1-scores across different folds suggests that these biologically inspired adjustments do not simply introduce noise, but actually capture underlying patterns in how specific ADRs relate to different structural and pharmacological drug profiles.

### 3.3. Limitations

Despite the promising results, several limitations must be acknowledged:

- Data Imbalance: Although the weighted strategy mitigated the effects of low prevalence, the intrinsic scarcity of data for certain ADRs remains a challenge for achieving high precision.
- Computational Complexity: Integrating multiple ensemble layers and hybrid biological rules increases the computational overhead compared to simpler, linear models.
- Generalizability: The current study focused on four specific disorder categories (RSBD, ELD, ED, BLSD). The performance of these strategies on a broader spectrum of multi-organ adverse reactions remains to be validated.
- Limitations of External Validation: While this study incorporates external validation using the real-world FAERS database, certain limitations remain. Specifically, the external mapping relied on keyword searches rather than the official MedDRA dictionary due to data access constraints, which may introduce mild classification noise. Future work will focus on integrating full MedDRA hierarchical coding to further refine real-world adverse event tracking.
- Limited Error Analysis: Although the quantitative evaluation demonstrated competitive performance, a detailed error analysis was not conducted. Future work should investigate the types of misclassifications, false positive and false negative patterns, and the biological characteristics associated with incorrectly predicted ADRs in order to further improve model interpretability and reliability.

### 3.4. Future Work

To build upon these findings, future research should focus on:

- Dynamic Thresholding: Developing an automated system that adjusts thresholds in real-time based on the incoming data stream’s imbalance ratio.
- Cross-Domain Validation: Applying this hybrid framework to other medical datasets, such as genomic markers or real-world evidence from Electronic Health Records (EHR), to test its robustness.
- Explainability (XAI): Integrating Explainable AI techniques to help clinicians understand why a specific biological weight was assigned to a prediction, thereby increasing trust in the model’s output. Recent pharmacovigilance research has demonstrated the value of explainable machine learning approaches, including SHAP-based analyses, for identifying influential predictors and improving the transparency of safety-signal assessment [27,28].

## 4. Materials and Methods

### 4.1. Data

We used the same dataset as in our previous studies [7,8], extracted from the Side Effect Resource database (SIDER). SIDER [29] is a comprehensive database that contains information about the side effects of drugs and marketed medicines, extracted from public documents and package inserts. This specific dataset, retrieved using the DeepChem (2.8.1.dev version) library [30], contains 1427 approved drugs, with ADRs that were text-mined from drug package inserts and grouped into 27 system organ classes (SOCs), following the Medical Dictionary for Regulatory Activities (MedDRA) classification. DeepChem is a software toolkit used to build artificial intelligence models for medicine and chemistry; it hosts and provides a clean, ready-to-use version of the SIDER dataset [30]. The MedDRA classification is a hierarchical system that codes medical terms in a standardized way, and this hierarchy is based on five levels [31]. The levels are System Organ Class (SOC), High-Level Group Term, High-Level Term, Preferred Term, and Lowest Level Term, with each level becoming more specific. This classification facilitates the entry, retrieval, and analysis of ADR and medical data across different organizations and countries.

The ADRs in the dataset are grouped into 27 SOCs, and this level of classification represents the highest and broadest level, grouping medical conditions by organ system, such as “Gastrointestinal disorders” or “Cardiac disorders”. Of all 27 SOCs, the models in this study were trained on 4 SOCs: Reproductive system and breast disorders (RSBD), ear and labyrinth disorders (ELD), eye disorders (ED) and blood and lymphatic system disorders (BLSD).

These four SOCs were selected because they include clinically significant ADRs with potentially severe and irreversible consequences, such as vision loss, hearing impairment, hematological abnormalities, or reproductive complications. In addition, these SOCs contain sufficiently representative ADR samples for machine learning analysis. They also provide diversity across multiple physiological systems, enabling a broader evaluation of the predictive models.

To further justify this selection against the broader dataset, Table 5 presents a comparative overview of the target SOCs alongside high-prevalence categories excluded from this study. Dominant categories such as gastrointestinal disorders (>85% prevalence) or nervous system disorders (>80% prevalence) appear across most drugs. However, they are skewed toward transient, low-severity side effects (e.g., nausea, headache [31]) which lower their clinical urgency for predictive modelling.

In contrast, the four selected SOCs offer a balance between high clinical impact (irreversible sensory, hematological, or endocrine damage) and sufficient dataset representation for machine learning convergence, with prevalence values ranging ~47% to ~62%.

RSBD-related ADRs may include changes in hormonal balance, menstrual irregularities, fertility issues, or breast changes that are causally linked to drug exposure. Some drugs can act as endocrine disruptors, and early prediction helps guide safer therapy and reduce long-term harm. Predicting these ADRs is especially critical in women of childbearing age and during pregnancy [32].

ELD-related ADRs refer to adverse effects on the auditory and vestibular systems, including hearing loss, ringing in the ears, dizziness, and impaired balance. Hearing and balance are vital sensory functions, and the resulting damage can be permanent. Prediction aids in risk stratification and monitoring, allowing clinicians to choose safer alternatives to avoid permanent auditory damage [33].

ED-related ADRs refer to adverse effects on any part of the eye or visual system, such as changes in vision, ocular inflammation, toxic effects on the retina or optic nerve, or other structural damage. The eye is highly sensitive to toxic substances, so these ADRs can rapidly lead to visual impairment or loss. Some ADRs are initially and only become apparent after significant damage has occurred. Predicting ocular ADR risk can help clinicians choose medications with safer ophthalmic profiles, monitor patients more closely, and prevent vision loss [34].

BLSD-related ADRs include adverse effects on the blood and lymphatic cells and organs, such as anemia, neutropenia, leukopenia, and other cytopenias. These conditions arise when medications interfere with hematopoiesis (blood cell production) or cause destruction of blood or lymphatic cells. Hematologic ADRs can be severe or life-threatening, leading to infections, bleeding, fatigue, or organ dysfunction. Predicting these ADRs enables clinicians to adjust treatment early, monitor blood counts, and prevent critical complications before they occur [35].

The dataset contains 28 features, 27 of them being the MedDRA SOC, having binary values (0 or 1) and the remaining feature, labelled SMILES, refers to the Simplified Molecular Input Line Entry System. SMILES [36] is a standardized way to represent a chemical structure as a text string, each string encoding the atoms, bonds, connectivity and, sometimes, stereochemistry of a molecule, making it easy for computational analyses, cheminformatics and ML models. Figure 13 presents the number of positives vs. negatives for each label and the relative balance in percentage between positive and negative examples. Table 6 presents a snippet of the SIDER dataset, where SMILES is the representation of the molecule, 0 value represents a negative outcome, meaning the molecule does not cause that specific ADR, and 1 value represents a positive outcome, meaning the molecule does cause that specific ADR.

### 4.2. Features

To generate features for the ML training process, we converted the SMILES notations into chemical fingerprints and molecular descriptors [7,8] using the RDKit library. Chemical fingerprints [37] are computational representations that encode the structural features of a molecule as a fixed-length bit vector, allowing materials, biological samples, or products to be identified and compared.

The fingerprints that were chosen for this research are: RDKit Fingerprint, Layered Fingerprint, Molecular Access System keys and Avalon Fingerprint.

The RDKit Fingerprint [38] is a 2D path-based molecular fingerprint, computed by enumerating all linear and branched atom and bond paths up to a certain length and hashing them into a bit vector. This fingerprint is useful because it captures the topological substructure information present in a molecule and is essential for encoding chemical substructure in a fixed-length vector.

Layered fingerprints [39] are a substructure or path-based representation that encodes fragments at different levels of structural complexity, organizing features into layers. They provide a hierarchical encoding of structural information, capturing details from simple to more complex substructures, and this multi-level detail can improve model discrimination, especially for tasks such as bioactivity prediction.

Molecular Access System keys [40] are a predefined set of structural keys, where each bit corresponds to the presence or absence of a specific chemical pattern. They are interpretable because each bit has a known chemical meaning, and they are well suited for fast similarity searching, clustering, and classification, especially when interpretability of chemical features is important.

Avalon fingerprints [40] are substructure key-based fingerprints originally developed for rapid substructure matching and screening. Unlike circular fingerprints, Avalon uses a larger set of structural features designed for efficient similarity and substructure searches in large chemical databases. Avalon performs well in classification and similarity tasks because it captures diverse structural patterns beyond simple rings or small fragments.

Table 7 presents a brief comparison summary of molecular fingerprints:

The molecular descriptors that we used for this research are Number of Hydrogen Bond Donors (NumHDonors), Number of Hydrogen Bond Acceptors (NumHAcceptors), Topological Polar Surface Area (TPSA), Log of Octanol/Water Partition Coefficient (MolLogP) and Number of Rotatable Bonds (NumRotatableBonds).

The NumHDonors counts the number of groups in a molecule capable of donating a hydrogen bond. Hydrogen bonds strongly influence molecular interactions with biological targets, solubility and membrane permeability [41].

The NumHAcceptors counts atoms in a molecule that can accept hydrogen bonds. Like donors, acceptors influence hydrogen bonding capacity, which affects solubility, permeability, bioavailability and binding affinity to targets [41].

TPSA measures the surface area contributed by polar atoms on a 2D molecular structure [42]. TPSA is strongly correlated with membrane permeability, oral bioavailability and transport properties such as blood–brain barrier penetration.

MolLogP estimates the partition coefficient between octanol and water which is a measure of a molecule’s lipophilicity [41]. Lipophilicity is fundamental to absorption, distribution, membrane permeability and toxicity.

NumRotatableBonds counts the non-ring single bonds that allow free rotation, reflecting the flexibility of a molecule. Molecular flexibility influences conformational entropy, target binding and pharmacokinetic properties [42].

Table 8 presents a brief comparison summary of molecular descriptors.

Several other common descriptors, such as Molecular Weight (MW) [7,8] and Heavy Atom Count, are frequently used in drug discovery; however, we deliberately excluded them to maintain a high signal-to-noise ratio. In our preliminary analysis, we found that MW was highly redundant, because its structural implications were already implicitly captured by the path-based RDKit fingerprints. Similarly, we opted not to include Molar Refractivity or simple Ring Counts. The former often correlates too closely with LogP, potentially leading to multi-collinearity issues that can confuse models like SVM. As for ring systems, our chosen Avalon and Layered fingerprints already provide a much more granular view of cyclic structures than a simple count could offer. By streamlining our feature set to the most impactful biophysical properties (such as TPSA and H-bond capacity), we reduced the risk of overfitting and ensured that our ensemble model focuses on the true drivers of adverse drug reactions.

### 4.3. Models Involved in the Research

In ML, an ensemble model is a technique that combines multiple individual models to produce a stronger overall model with better performance, stability and generalization than any single model alone. Recent studies [43] have demonstrated the utility of ensemble learning strategies, including averaging, voting, stacking, for improving the robustness of molecular activity prediction. There are multiple ensemble-learning types, for example: Bagging, Boosting, Stacking, and Voting. Voting is one of the simplest ensemble strategies: it combines predictions from multiple models (usually classification models) and selects the final output based on a voting rule. There are two main voting types: hard voting, where each model votes for one class and the class receiving the most votes is selected, and soft voting, where the class with the highest average predicted probability across models is selected.

The baseline model in this study is a hybrid ensemble combining three different supervised classifiers: RF, SVM and XGB. Each model is set up to handle multiple labels at once, since a compound can trigger several ADRs. To make the final prediction, we average the probability outputs from all three models through soft voting. RF brings robustness and handles complex patterns well, SVM captures subtle, non-linear relationships and XGB boosts performance by focusing on the most challenging cases. By combining them, the ensemble benefits from the strengths of each algorithm, giving a more reliable and balanced prediction.

We considered using the soft voting ensemble because it combines simplicity, robustness, and improved accuracy, relying not only on the predictions of the individual models, but also on how confident each model is. For example, in hard voting, each model votes for a class, whereas in soft voting, each model provides a probability for each class. Soft voting is easy to implement and can significantly improve results compared to using a single model. It also reduces the risk that one weak model dominates the decision and makes the ensemble less sensitive to noise and random fluctuations in the data.

### 4.4. Model Architecture and Technical Configuration

The models were configured to balance predictive power with generalization, specifically:

- RF: We utilized an ensemble of 500 decision trees with a maximum depth of 6. This depth was deliberately constrained to prevent overfitting on molecular fingerprints, ensuring the model captures broad structural patterns rather than memorizing the training set.
- SVM: Implemented with a Radial Basis Function (RBF) kernel, which is essential for capturing the non-linear boundaries inherent in high-dimensional chemical spaces. We enabled probability estimates to facilitate the soft-voting mechanism, using a standard regularization parameter (*C* = 1.0) and a scaled gamma.
- XGB: This model was tuned with a learning rate of 0.05 and 500 estimators. By using a relatively low learning rate and a maximum depth of 6, the model iteratively corrects the residuals of previous trees, focusing on the hard-to-classify molecular structures that the RF or SVM might overlook.

To consolidate the findings, we implemented a soft voting (probability averaging) mechanism. Instead of a simple majority vote, the ensemble calculates the mean of the predicted probabilities (*P*) from all three models. The ensemble prediction is defined in Equation (1):

(1) $$P_{ensemble}=\frac{P_{RF}+P_{SVM}+P_{XGB}}{3}$$

A classification threshold of 0.5 was applied to these average probabilities to generate the final binary predictions. Given the medical context of this research, our evaluation prioritized recall-oriented metrics. While Precision is important, maximizing Recall was prioritized to ensure that potential adverse reactions are not missed (minimizing false negatives), which is a critical safety requirement in pharmacovigilance.

### 4.5. Models’ Training

For model training we combined the generated fingerprints, RDKit fingerprints, Layered fingerprints, Molecular Access System keys and Avalon fingerprints, with the molecular descriptors, NumHDonors, NumHAcceptors, TPSA, MolLogP and NumRotatableBonds. To improve predictions for rare ADRs, we applied two biologically inspired adjustments: balanced thresholds [44] and weighted predictions [45].

For the balanced thresholds strategy, for each label, we adjusted the probability cut-off based on how common the reaction is in the training data. Less common reactions had lower thresholds to improve recall. Mathematically, for each target label *j*, the decision threshold *T_j_* was derived from the training prevalence *p_j_* using a non-linear decay function bounded between 0.20 and 0.50, defined in Equation (2):

(2) $$T_{j}=clip(0.50\cdot {\left(1-\;p_{j}\right)}^{0.6},0.20,0.50)$$

A test sample was assigned a positive prediction if *P_ensemble_* ≥ T_j_.

For the weighted predictions strategy, we scaled the predicted probabilities by the inverse frequency of each label, giving more weight to rare reactions. Specifically, we incorporated a smoothing term ε = 0.01 to prevent division by zero, scaling the ensemble probabilities before applying the standard 0.50 decision threshold, defined in Equation (3):

(3) $$w_{j}=\frac{1}{p_{j}+0.01},\;P_{weighted,\;j}=clip(P_{ensemble,\;j}\;\cdot \;w_{j},\;0,\;1)$$

This helps the model pay more attention to ADRs that occur less frequently but are clinically important. The use of balanced thresholds and weighted predictions is inspired by biological principles, reflecting the fact that some ADRs are rare but clinically important, while others occur more frequently with less severity. By adjusting the probability thresholds and weighting predictions according to each reaction, the model prioritizes detection of less common but critical ADRs, similar to how biological systems are tuned to respond to significant signals over routine events [44,45].

To prevent data leakage and ensure an unbiased evaluation, all parameters required for the balanced thresholds and weighted predictions strategies were calculated exclusively on the training folds of each cross-validation iteration and then applied independently to the corresponding validation fold.

To ensure robust statistical power and eliminate dependency on a single data partition, we evaluated the model using a 5 × 5 repeated k-fold cross validation strategy (5 repetitions of 5-fold cross validation, totaling 25 evaluation rounds). To improve the comparability and reliability of the probability estimates used in the soft-voting ensemble, probability calibration using sigmoid (Platt) scaling was applied to the RF and XGB classifiers through 3-fold internal cross-validation. For the SVM classifier, probability estimates were enabled using the built-in probability estimation procedure of SVC. The resulting class probabilities were subsequently averaged to obtain the ensemble prediction. Across each iteration, the dataset was partitioned, training on four folds and testing on the remaining one.

Platt scaling [46] fits a parametric logistic regression model to map raw model decision scores into reliable posterior probabilities, defined as in Equation (4):

(4) $$P\left(y=1|f\right)=\frac{1}{1+exp\left(A\cdot f+B\right)}$$

where f represents the raw decision value, and A and B are parameters optimized via maximum likelihood on the training dataset.

Figure 14 presents the entire training and evaluation process.

### 4.6. Feature Encoding, Preprocessing and Hyperparameter Selection

Since the input space relies on a mix of structural fingerprints and numerical molecular properties, preprocessing was adjusted according to feature type. The bit-vectors (RDKit, Layered, MACCS and Avalon) were already encoded as binary variables (∈ {0, 1}) so no further transformations were applied on them.

For the continuous descriptors (NumHDonors, NumHAcceptors, TPSA, MolLogP, and NumRotatableBonds), we decided against using standard normalization or min-max scaling, in order to keep the physical values directly interpretable. This choice had no adverse effect on model performance, given the specific algorithms chosen: (1) tree-based models like RF and XGB make decisions via feature splits and are completely invariant to monotonic scaling; (2) the SVM model with an RBF kernel used the default scaled gamma parameter, which automatically accounts for baseline variance differences across features without needing prior scaling.

To control model complexity and reduce the risk of overfitting on the combined feature set, fixed hyperparameter configurations were used for each base model. For RF, 500 trees with a maximum depth of 6 were used. The ensemble structure provides averaging across multiple trees, while the depth constraint limits the complexity of individual trees [47]. XGB was configured with 500 boosting rounds, a maximum depth of 6, and a learning rate of 0.05, using a binary log-loss objective. A maximum depth of 6 is the default value documented for XGB, while the learning rate acts as a shrinkage parameter that makes boosting updates more conservative and can help prevent overfitting [48]. For SVM, an RBF kernel was used with C = 1.0 and gamma = ‘scale’, which correspond to the standard SVC defaults in scikit-learn; C controls the regularization strength, while gamma is automatically scaled according to the number and variance of the input features [49]. Finally, sigmoid calibration (Platt scaling) was applied to obtain calibrated probability estimates required for the soft-voting procedure.

### 4.7. Performance Metrics

To evaluate the performance of the models, we used the recall, precision, F1-score and Area Under the Precision-Recall Curve (AUC-PR) which are widely used metrics in multi-label and imbalanced classification problems, such as predicting ADRs.

Recall [50] measures the proportion of actual positive cases that the model correctly identifies. Recall is defined in Equation (5):

(5) $$Recall=\;\frac{TP}{TP+FN}$$

where TP is True Positive and FN is False Negative. According to Equation (5), recall decreases as the number of false negatives increases. High recall ensures that the most true ADRs are detected, which is critical in pharmacovigilance, where missing a serious ADR can have severe consequences.

Precision [50] measures the proportion of predicted positive cases that are actually correct. Precision is defined in Equation (6):

(6) $$Precision=\;\frac{TP}{TP+FP}$$

where FP is False Positive. As shown in Equation (6), precision is inversely proportional to the number of false positives. High precision ensures that the predicted ADRs are reliable, reducing false alarms and is also important for minimizing unnecessary follow-up, overestimation of drug risks or clinical workload.

F1-score [50] is the harmonic mean of precision and recall, providing a balance between detecting true positives and avoiding false positives. The F1-score is defined in Equation (7):

(7) $$F1=2\frac{Precision\cdot Recall}{Precision+Recall}$$

where Equation (7) shows that the F1-score balances both precision and recall. F1-score is especially useful in imbalanced multi-label datasets, where ADRs may be rare. It balances the trade-off between recall and precision, giving a single summary measure of model performance. Using F1 ensures that the model is not only sensitive but also accurate in its predictions, which is crucial for pharmacological safety assessments.

AUC-PR [50] is a metric that evaluates how well a classification model performs across all possible decision thresholds by measuring the trade-off between precision and recall. The AUC-PR is defined in Equation (8):

(8) $$AUC-PR=\int_{0}^{1}P\left(R\right)dR$$

where P(R) is the precision function evaluated at a given recall level R.

These metrics are standard in cheminformatics and pharmacovigilance studies because they reflect both the safety and reliability of predictions.

Precision, recall, F1-score and AUC-PR were selected as the primary evaluation metrics because ADR prediction represents a highly imbalanced multi-label classification problem, where positive ADR instances are significantly rarer than negative samples. Other metrics frequently used in machine learning research were explicitly excluded from this study due to structural or conceptual limitations when applied to sparse pharmacovigilance data:

- Strict multi-label metrics (Exact Match Ratio): Also known as Subset Accuracy, the Exact Match Ratio requires the model to predict the entire set of labels identically to the ground truth for a given instance. In large-scale ADR prediction involving hundreds of potential side effects, this metric is overly punitive and clinically irrelevant; a model that correctly predicts 49 out of 50 severe ADRs for a compound would receive a score of zero, completely masking its high clinical utility.
- Sparsity-biased metrics (Hamming Loss): Hamming Loss measures the fraction of misclassified instance-label pairs. In sparse datasets where the vast majority of labels are negative (i.e., a drug does not cause most of the listed ADRs), a naive baseline model that predicts “no ADR” for everything will automatically achieve a near-zero Hamming Loss. Therefore, this metric reflects the sparsity of the dataset rather than the model’s actual ability to discover active ADRs.
- Averaging techniques (Macro-F1 vs. Micro-F1): Micro-F1 aggregates the contributions of all classes to compute the global average, which in ADR datasets causes the metric to be heavily dominated by a few highly frequent, common side effects (e.g., headaches or nausea), obscuring model performance on rare, critical toxicities. Conversely, Macro-F1 averages the F1-scores of each class independently, making it overly sensitive to extreme data scarcity where a single true positive or false positive in a rare class can cause radical, unrepresentative fluctuations in the final score.

By focusing strictly on Precision, Recall, the instance-level F1-score, and AUC-PR this study ensures that the evaluation directly reflects the safety, reliability, and clinical relevance of the predictions in a clear and interpretable manner.

## 5. Conclusions

In this study, we evaluated a hybrid ensemble model for predicting adverse drug reactions and explored biologically inspired strategies, including balanced thresholds and weighted predictions. The main motivation for creating the proposed ensemble model by combining Random Forest, Extreme Gradient Boosting, and Support Vector Machine was to combine the strengths of different machine-learning algorithms so that the final model can make more robust and accurate predictions than a single model. Our results show that these strategies can significantly improve model sensitivity, particularly for low-prevalence ADRs, without drastically compromising overall predictive performance. Weighted predictions were especially effective at detecting rare ADRs, while balanced thresholds enhanced recall for more common reactions. The baseline hybrid model maintained higher precision, highlighting the trade-off between sensitivity and specificity. Overall, integrating biological knowledge into predictive models provides a practical and effective approach to improve ADR detection, offering a flexible framework that can be adapted to different clinical priorities.

## Figures and Tables

**Figure 1:**
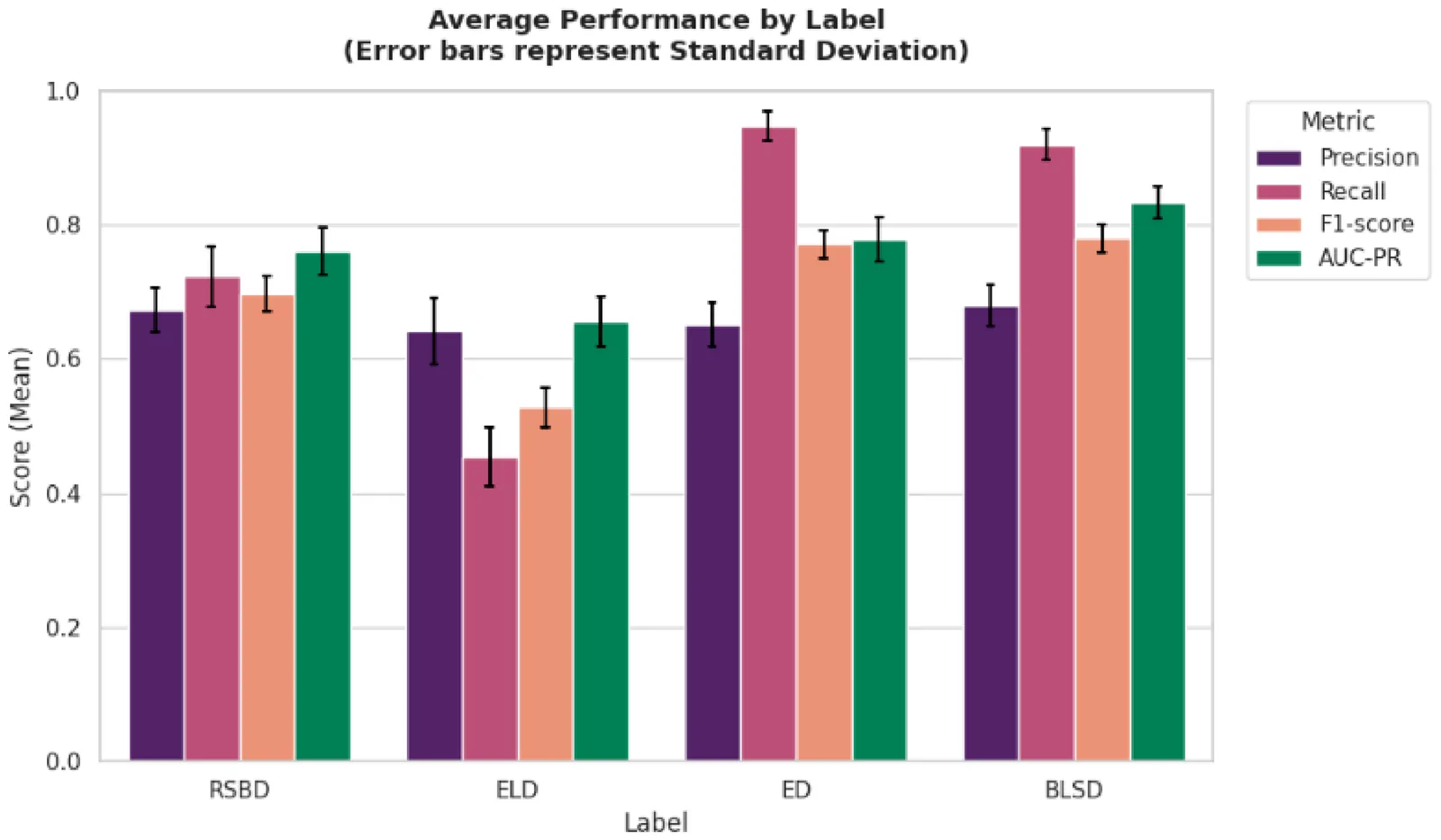
Average performance of the baseline hybrid model across 25 repeated cross-validation iterations. Bars show mean Recall, Precision, F1-score, and AUC-PR for each SOC. Error bars represent the standard deviation across the 25 evaluation iterations.

**Figure 2:**
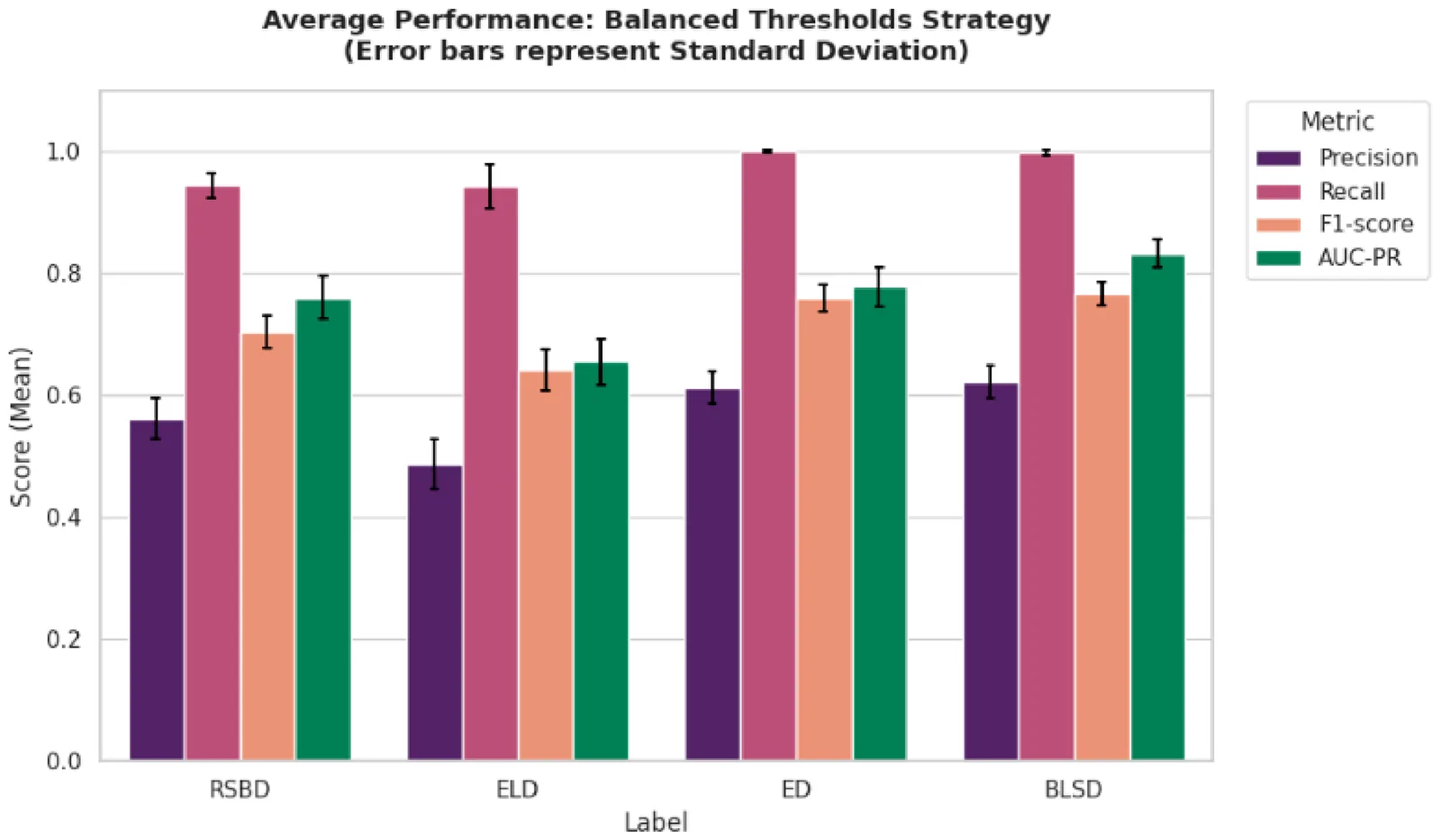
Average performance of the balanced thresholds strategy across 25 repeated cross-validation iterations. Bars show mean Recall, Precision, F1-score, and AUC-PR for each SOC. Error bars represent the standard deviation across the 25 evaluation iterations.

**Figure 3:**
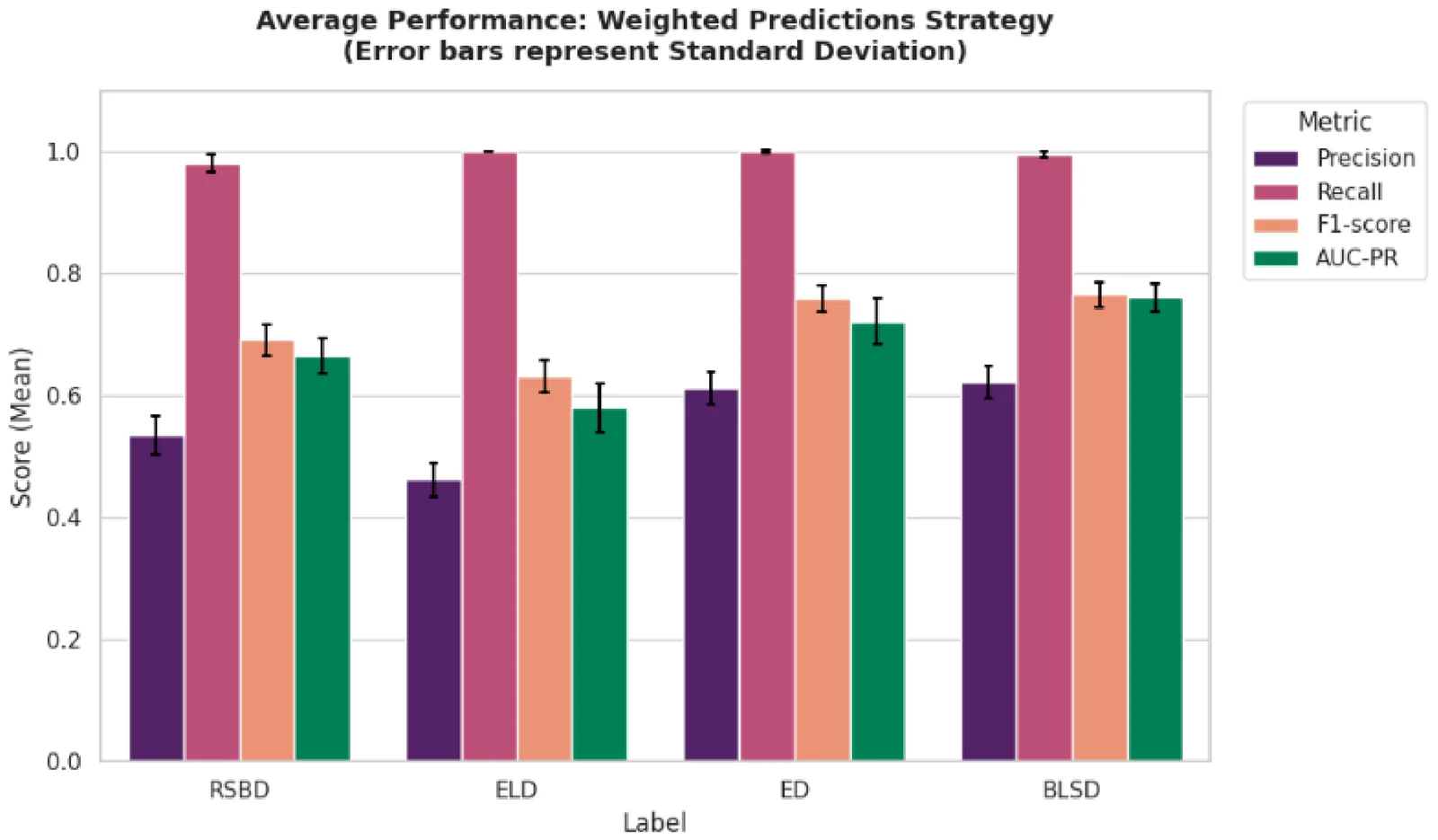
Average performance of the weighted predictions strategy across 25 repeated cross-validation iterations. Bars show mean Recall, Precision, F1-score, and AUC-PR for each SOC. Error bars represent the standard deviation across the 25 evaluation iterations.

**Figure 4:**
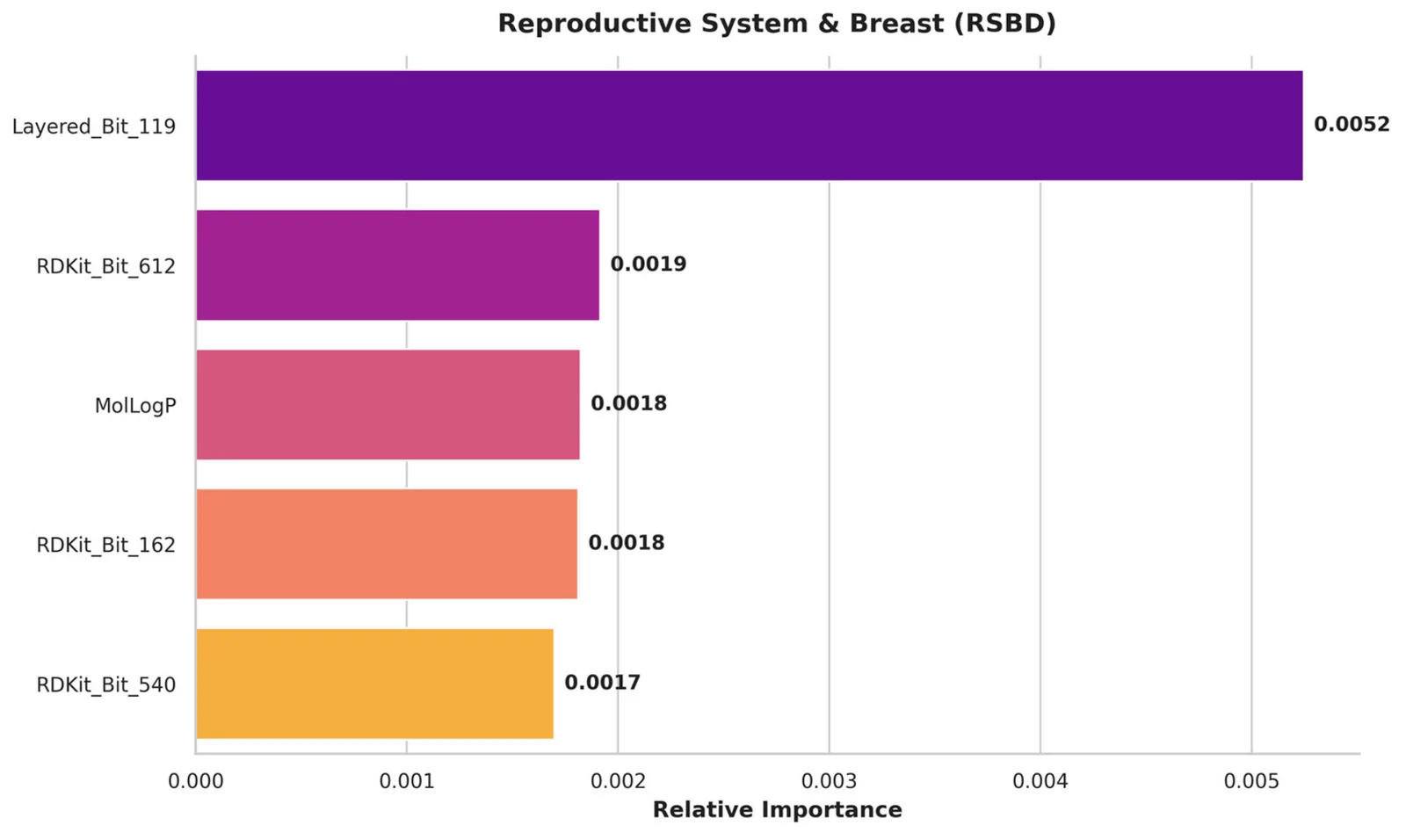
Model interpretability and top molecular descriptors for RSBD.

**Figure 5:**
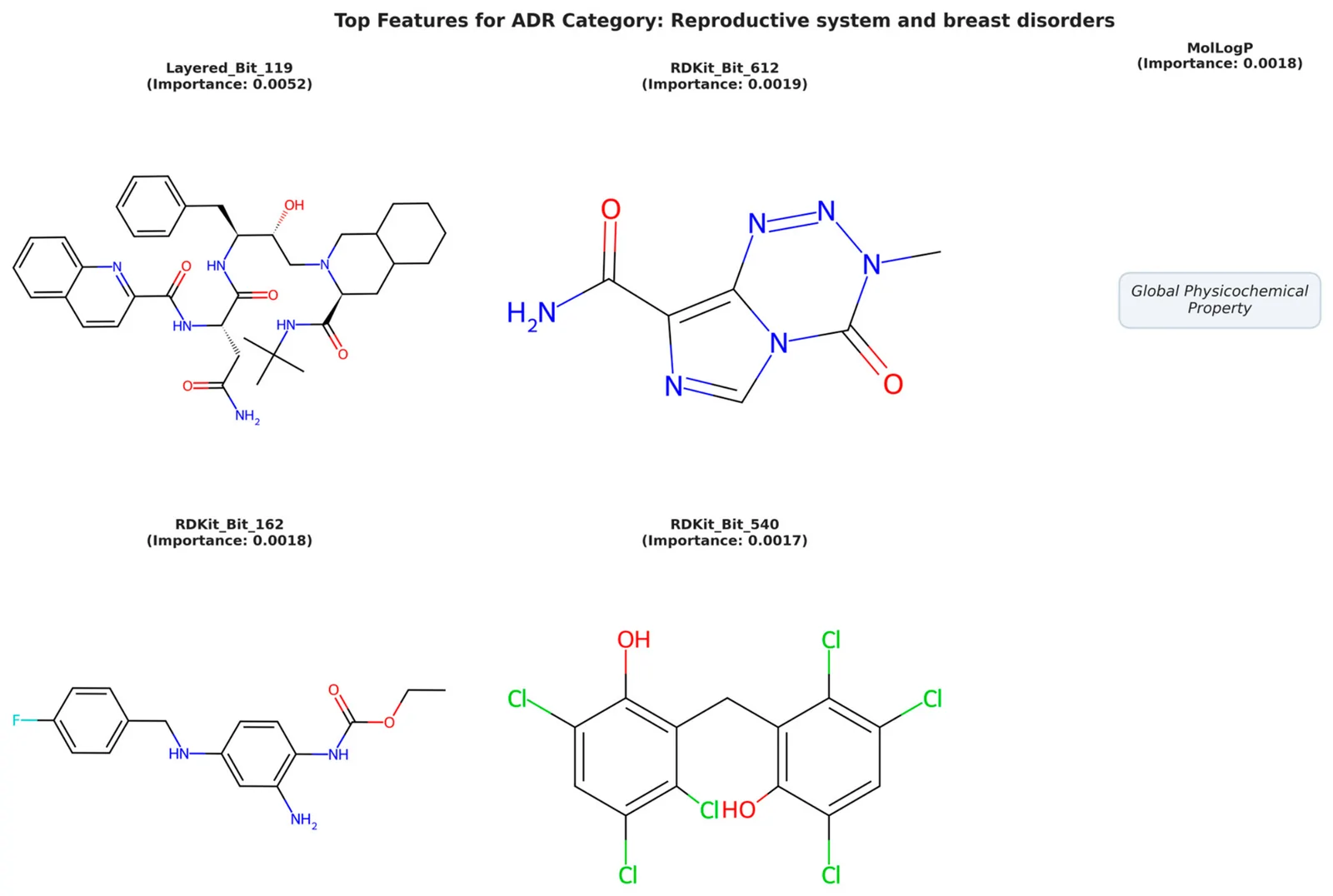
Top features and structural fragments for RSBD.

**Figure 6:**
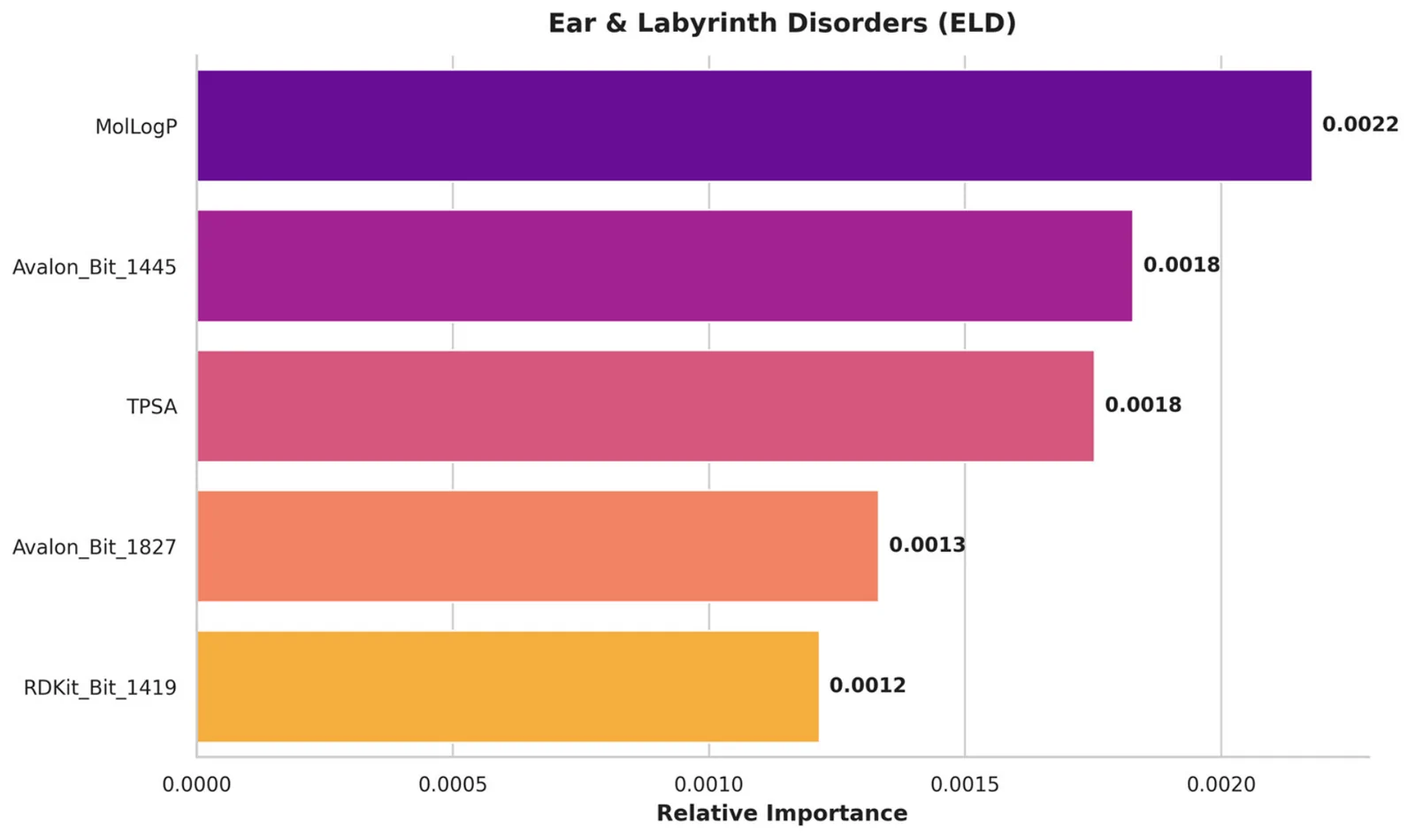
Model interpretability and top molecular descriptors for ELD.

**Figure 7:**
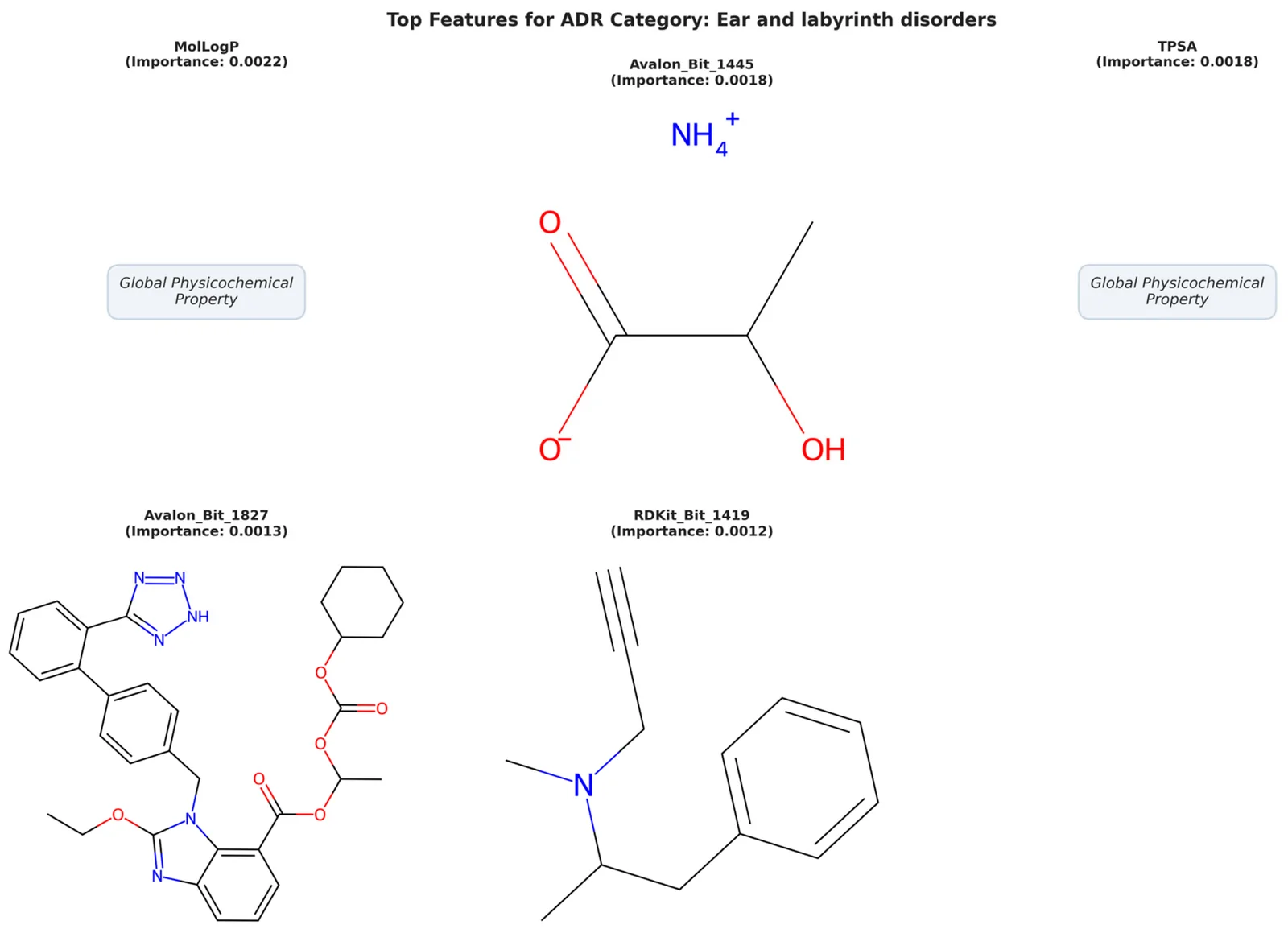
Top features and structural fragments for ELD.

**Figure 8:**
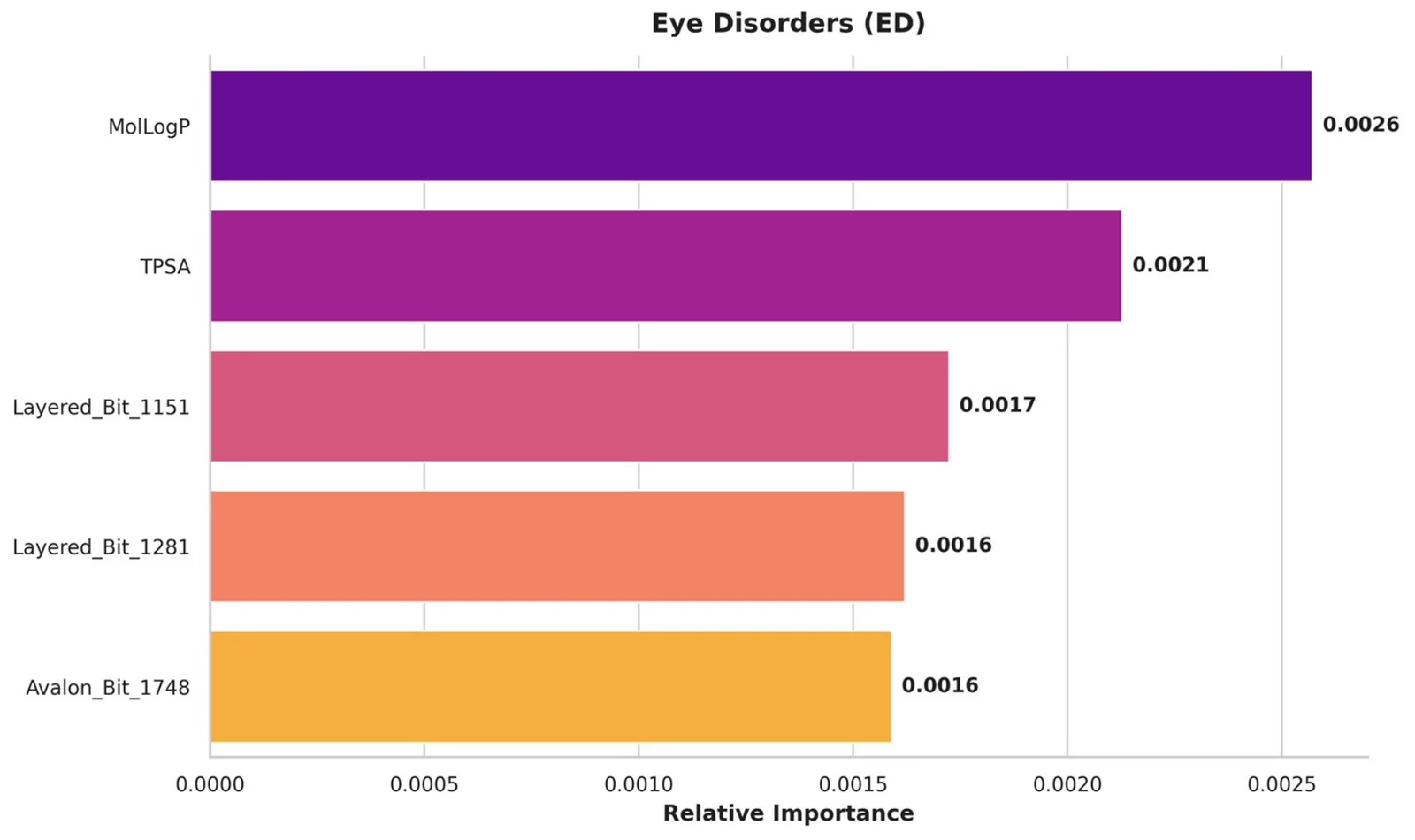
Model interpretability and top molecular descriptors for ED.

**Figure 9:**
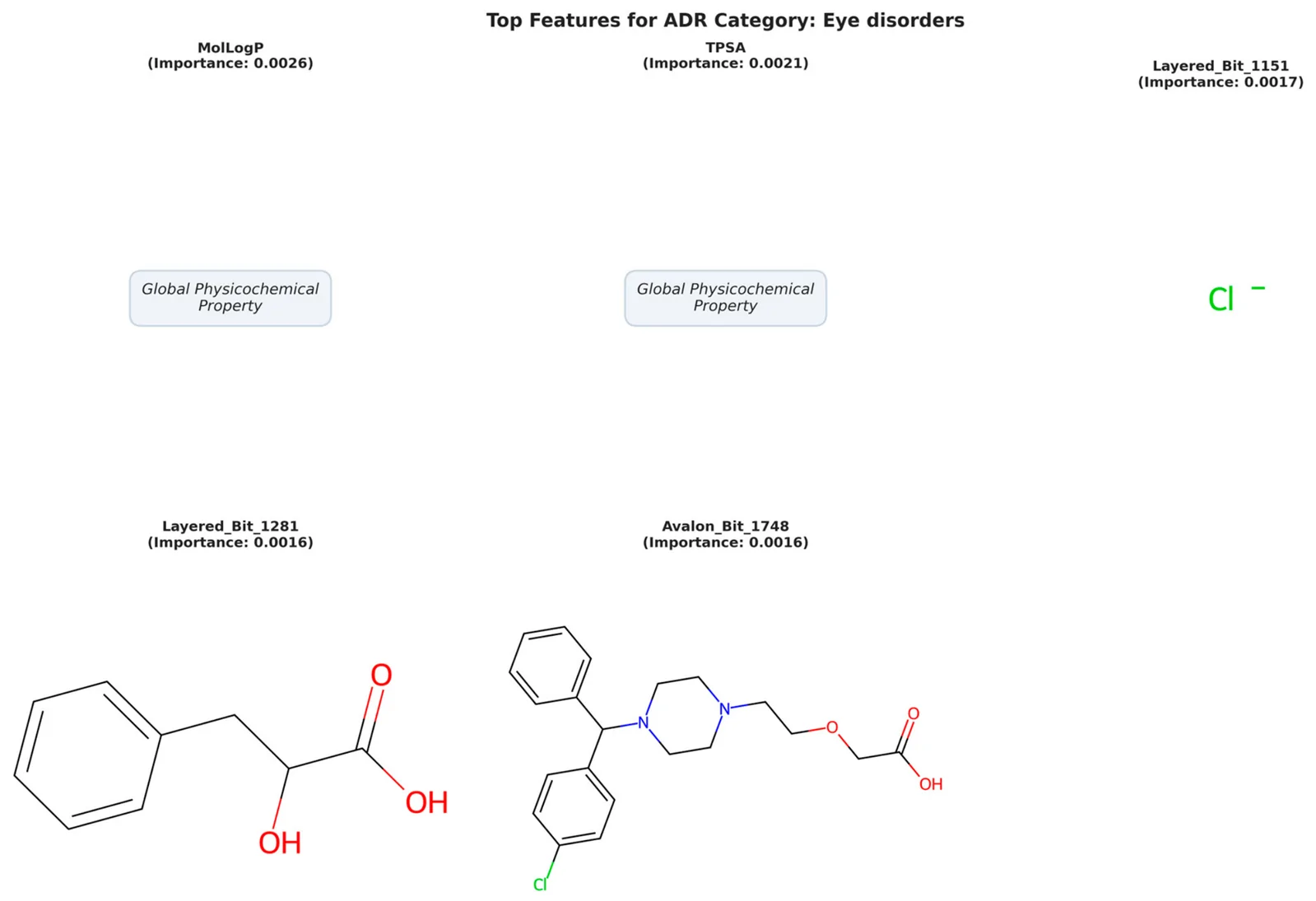
Top features and structural fragments for ED.

**Figure 10:**
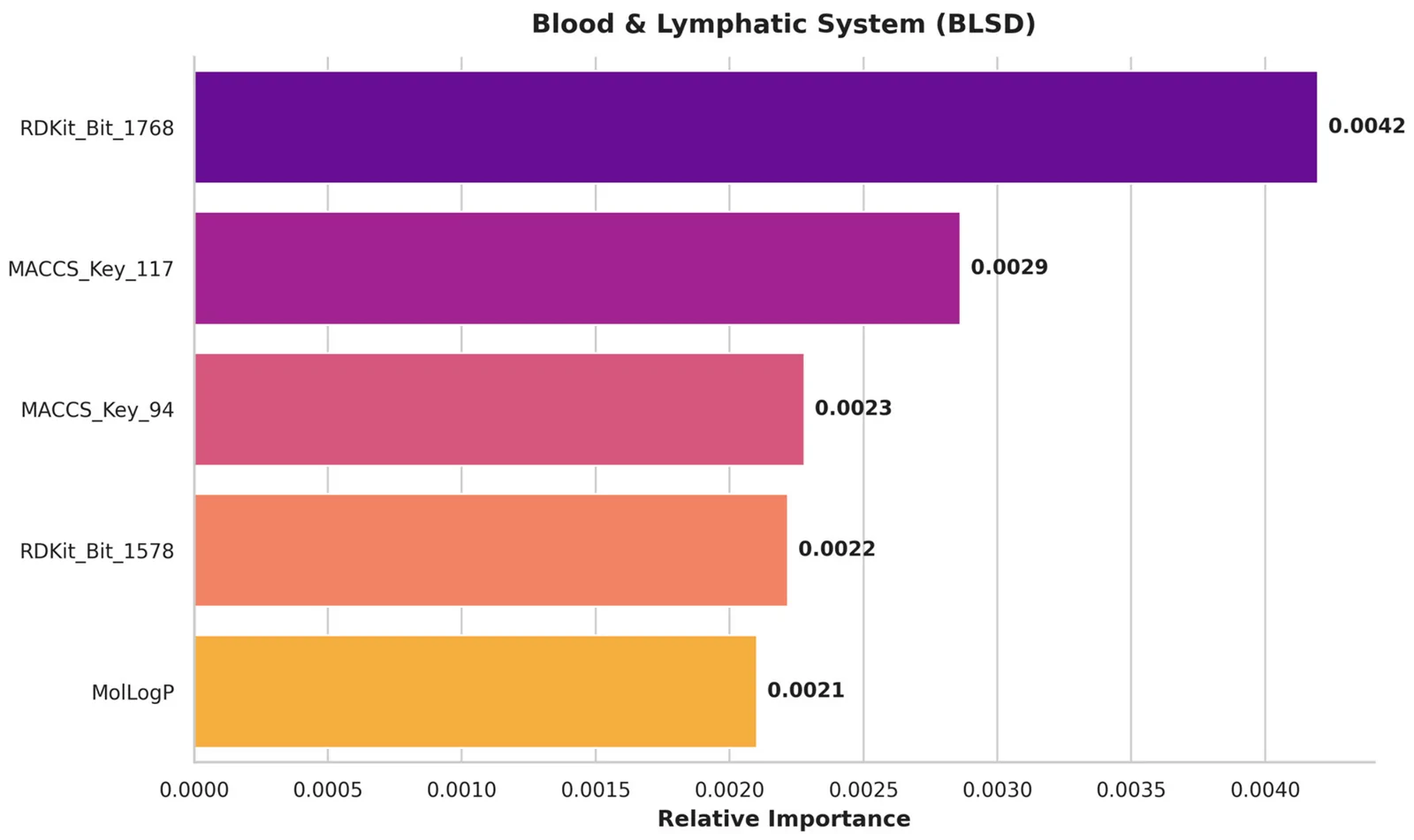
Model interpretability and top molecular descriptors for BLSD.

**Figure 11:**
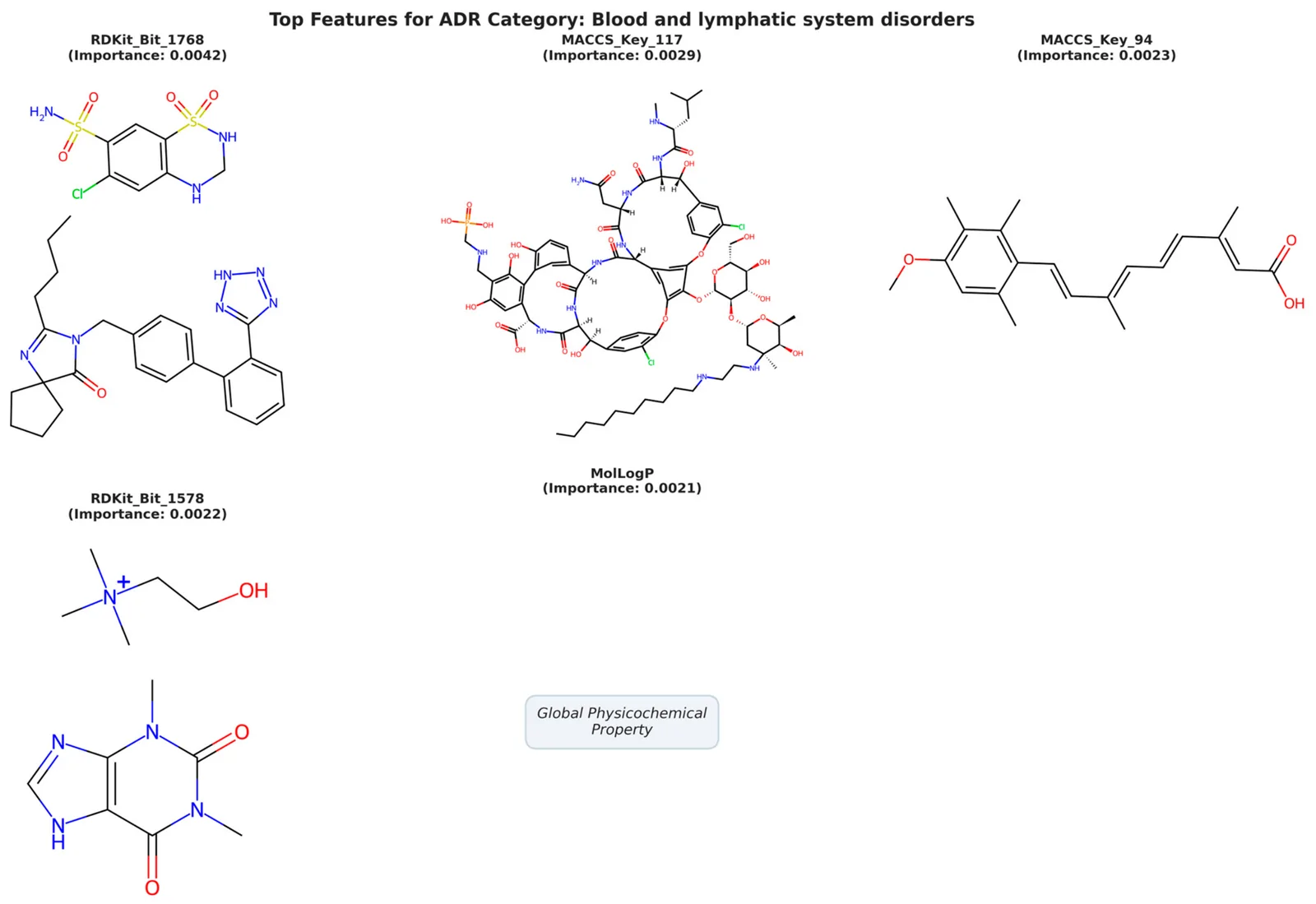
Top features and structural fragments for BLSD.

**Figure 12:**
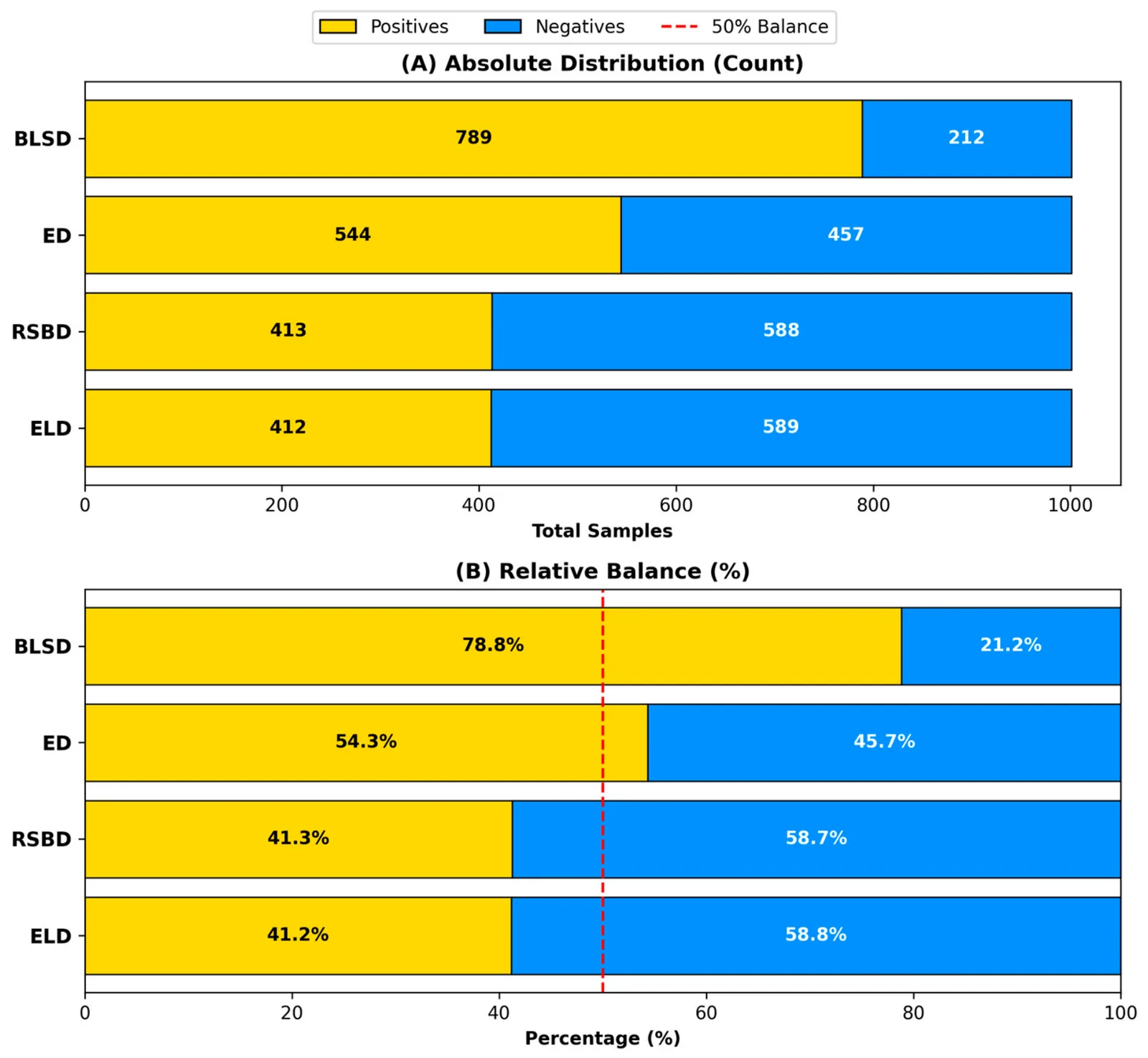
Number of positive vs. negative examples on FAERS dataset. (**A**) Absolute distribution showing the exact sample counts for each label, and (**B**) Relative balance illustrating the percentage proportions relative to a 50% threshold.

**Figure 13:**
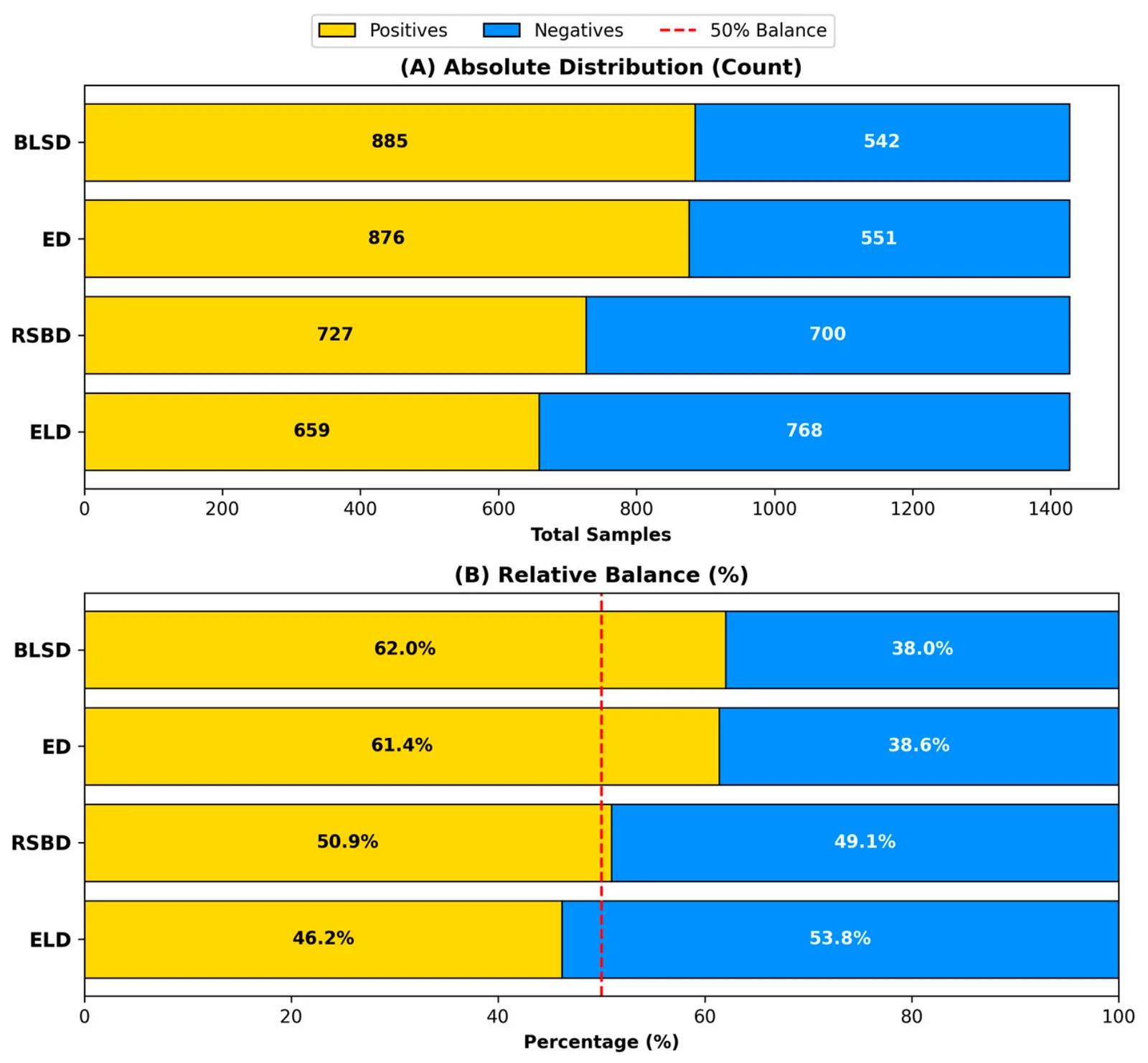
Number of positive vs. negative examples. (**A**) Absolute distribution showing the exact sample counts for each label, and (**B**) Relative balance illustrating the percentage proportions relative to a 50% threshold.

**Figure 14:**
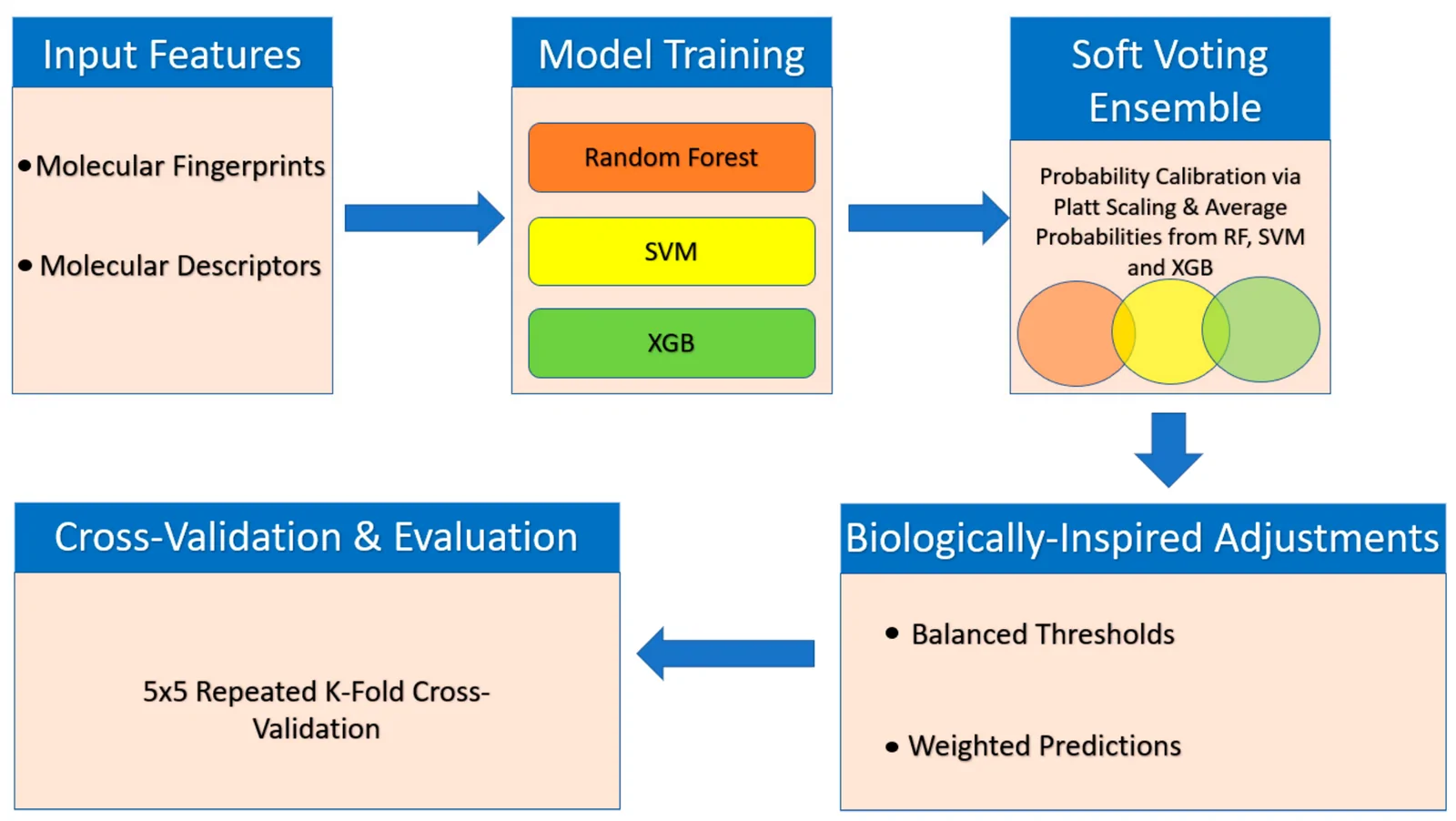
Training and evaluation process.

**Table 1:** Mean performance of the standalone classifiers across 25 repeated cross-validation iterations.

SOC	Model	Recall (Mean ± SD)	Precision (Mean ± SD)	F1-Score (Mean ± SD)	AUC-PR (Mean ± SD)
RSBD	RF	0.721 ± 0.066	0.657 ± 0.037	0.686 ± 0.044	0.755 ± 0.044
RSBD	SVM	0.640 ± 0.126	0.609 ± 0.067	0.618 ± 0.109	0.621 ± 0.050
RSBD	XGB	0.700 ± 0.068	0.677 ± 0.040	0.688 ± 0.049	0.764 ± 0.041
ELD	RF	0.520 ± 0.088	0.606 ± 0.054	0.556 ± 0.049	0.648 ± 0.041
ELD	SVM	0.253 ± 0.210	0.592 ± 0.234	0.312 ± 0.191	0.579 ± 0.067
ELD	XGB	0.521 ± 0.095	0.632 ± 0.046	0.568 ± 0.052	0.664 ± 0.039
ED	RF	0.905 ± 0.031	0.665 ± 0.038	0.766 ± 0.022	0.769 ± 0.041
ED	SVM	0.977 ± 0.013	0.623 ± 0.027	0.760 ± 0.019	0.738 ± 0.039
ED	XGB	0.905 ± 0.035	0.670 ±0.034	0.770 ± 0.023	0.778 ± 0.032
BLSD	RF	0.810 ± 0.035	0.706 ± 0.040	0.753 ± 0.031	0.818 ± 0.054
BLSD	SVM	0.976 ± 0.046	0.628 ± 0.028	0.763 ± 0.021	0.790 ± 0.036
BLSD	XGB	0.872 ± 0.030	0.700 ± 0.028	0.777 ± 0.020	0.828 ± 0.022

Note: Values are reported as mean ± standard deviation (SD) across 25 repeated cross-validation iterations. SOC, system organ class; RSBD, reproductive system and breast disorders; ELD, ear and labyrinth disorders; ED, eye disorders; BLSD, blood and lymphatic system disorders; RF, Random Forest; SVM, Support Vector Machine; XGB, Extreme Gradient Boosting; AUC-PR, area under the precision–recall curve.

**Table 2:** Wilcoxon Statistical Comparison Results.

Label	Metric	Compared Model	Hybrid Mean	Model Mean	Winner	*p*-Value	Significant
RSBD	Recall	RF	0.7224	0.7332	RF	0.052187	FALSE
RSBD	Precision	RF	0.6728	0.6616	Hybrid	0.002644	TRUE
RSBD	F1-score	RF	0.6964	0.6944	Hybrid	0.520112	FALSE
RSBD	AUC-PR	RF	0.7604	0.7608	RF	0.98914	FALSE
RSBD	Recall	SVM	0.7224	0.6652	Hybrid	6.40 × 10^−5^	TRUE
RSBD	Precision	SVM	0.6728	0.6172	Hybrid	1.60 × 10^−5^	TRUE
RSBD	F1-score	SVM	0.6964	0.6384	Hybrid	1.30 × 10^−5^	TRUE
RSBD	AUC-PR	SVM	0.7604	0.6252	Hybrid	1.30 × 10^−5^	TRUE
RSBD	Recall	XGB	0.7224	0.7128	Hybrid	0.153764	FALSE
RSBD	Precision	XGB	0.6728	0.6816	XGB	0.011267	TRUE
RSBD	F1-score	XGB	0.6964	0.6968	XGB	0.712884	FALSE
RSBD	AUC-PR	XGB	0.7604	0.7692	XGB	0.094791	FALSE
ELD	Recall	RF	0.454	0.502	RF	2.10 × 10^−5^	TRUE
ELD	Precision	RF	0.6416	0.6028	Hybrid	1.30 × 10^−5^	TRUE
ELD	F1-score	RF	0.528	0.5464	RF	0.0025	TRUE
ELD	AUC-PR	RF	0.6552	0.6432	Hybrid	8.80 × 10^−5^	TRUE
ELD	Recall	SVM	0.454	0.2172	Hybrid	1.30 × 10^−5^	TRUE
ELD	Precision	SVM	0.6416	0.5828	Hybrid	0.161632	FALSE
ELD	F1-score	SVM	0.528	0.2868	Hybrid	1.30 × 10^−5^	TRUE
ELD	AUC-PR	SVM	0.6552	0.5692	Hybrid	1.30 × 10^−5^	TRUE
ELD	Recall	XGB	0.454	0.5016	XGB	1.80 × 10^−5^	TRUE
ELD	Precision	XGB	0.6416	0.628	Hybrid	0.018511	TRUE
ELD	F1-score	XGB	0.528	0.5564	XGB	0.000358	TRUE
ELD	AUC-PR	XGB	0.6552	0.6604	XGB	0.420356	FALSE
ED	Recall	RF	0.946	0.9076	Hybrid	1.70 × 10^−5^	TRUE
ED	Precision	RF	0.65	0.6616	RF	0.000108	TRUE
ED	F1-score	RF	0.7704	0.7644	Hybrid	0.010603	TRUE
ED	AUC-PR	RF	0.7776	0.7636	Hybrid	1.40 × 10^−5^	TRUE
ED	Recall	SVM	0.946	0.976	SVM	1.30 × 10^−5^	TRUE
ED	Precision	SVM	0.65	0.6224	Hybrid	1.10 × 10^−5^	TRUE
ED	F1-score	SVM	0.7704	0.7592	Hybrid	8.70 × 10^−5^	TRUE
ED	AUC-PR	SVM	0.7776	0.7344	Hybrid	1.20 × 10^−5^	TRUE
ED	Recall	XGB	0.946	0.9048	Hybrid	2.70 × 10^−5^	TRUE
ED	Precision	XGB	0.65	0.6672	XGB	2.00 × 10^−5^	TRUE
ED	F1-score	XGB	0.7704	0.768	Hybrid	0.408435	FALSE
ED	AUC-PR	XGB	0.7776	0.7732	Hybrid	0.12042	FALSE
BLSD	Recall	RF	0.9192	0.814	Hybrid	1.30 × 10^−5^	TRUE
BLSD	Precision	RF	0.68	0.7128	RF	1.20 × 10^−5^	TRUE
BLSD	F1-score	RF	0.78	0.7592	Hybrid	1.20 × 10^−5^	TRUE
BLSD	AUC-PR	RF	0.8328	0.8288	Hybrid	0.002957	TRUE
BLSD	Recall	SVM	0.9192	0.9856	SVM	1.20 × 10^−5^	TRUE
BLSD	Precision	SVM	0.68	0.6256	Hybrid	1.30 × 10^−5^	TRUE
BLSD	F1-score	SVM	0.78	0.7652	Hybrid	0.000749	TRUE
BLSD	AUC-PR	SVM	0.8328	0.792	Hybrid	1.40 × 10^−5^	TRUE
BLSD	Recall	XGB	0.9192	0.874	Hybrid	1.30 × 10^−5^	TRUE
BLSD	Precision	XGB	0.68	0.7012	XGB	4.70 × 10^−5^	TRUE
BLSD	F1-score	XGB	0.78	0.778	Hybrid	0.901751	FALSE
BLSD	AUC-PR	XGB	0.8328	0.8288	Hybrid	0.094573	FALSE

**Table 3:** Comparative performance of the three modelling strategies across disorder categories.

Disorder	Metric	Baseline Hybrid Model	Balanced Thresholds	Weighted Predictions	Friedman *p*	Binomial *p*
RSBD	Recall	0.72	0.94	0.98	<0.0001	<0.0001
	Precision	0.67	0.56	0.54	<0.0001	1
	F1-score	0.70	0.70	0.69	0.0132	0.655
	AUC-PR	0.76	0.76	0.67	<0.0001	1
ELD	Recall	0.45	0.94	1.00	<0.0001	<0.0001
	Precision	0.64	0.49	0.46	<0.0001	1
	F1-score	0.53	0.64	0.63	<0.0001	<0.0001
	AUC-PR	0.66	0.66	0.58	<0.0001	1
ED	Recall	0.95	1.00	1.00	<0.0001	<0.0001
	Precision	0.65	0.61	0.61	<0.0001	1
	F1-score	0.77	0.76	0.76	<0.0001	1
	AUC-PR	0.78	0.78	0.72	<0.0001	1
BLSD	Recall	0.92	1.00	1.00	<0.0001	<0.0001
	Precision	0.68	0.62	0.62	<0.0001	1
	F1-score	0.78	0.77	0.77	<0.0001	1
	AUC-PR	0.83	0.83	0.76	<0.0001	1

**Table 4:** External validation performance of the baseline hybrid model, Balanced Thresholds strategy, and Weighted Predictions strategy on the FAERS dataset.

SOC	Strategy	Recall	Precision	F1-Score	AUC-PR
RSBD	Baseline hybrid	0.55	0.40	0.47	0.40
RSBD	Balanced thresholds	0.77	0.39	0.52	0.40
RSBD	Weighted predictions	1	0.38	0.55	0.39
ELD	Baseline hybrid	0.34	0.38	0.36	0.37
ELD	Balanced thresholds	0.78	0.36	0.50	0.37
ELD	Weighted predictions	1.00	0.35	0.52	0.37
ED	Baseline hybrid	0.90	0.52	0.65	0.55
ED	Balanced thresholds	1.00	0.50	0.66	0.80
ED	Weighted predictions	1.00	0.50	0.67	0.51
BLSD	Baseline hybrid	0.82	0.79	0.80	0.80
BLSD	Balanced thresholds	1.00	0.78	0.88	0.81
BLSD	Weighted predictions	0.97	0.78	0.86	0.81

**Table 5:** SIDER dataset prevalence.

SOC	Count (n)	Prevalence (%)
Skin and subcutaneous tissue disorders	1318	92.36
Nervous system disorders	1304	91.38
Gastrointestinal disorders	1298	90.96
General disorders and administration site conditions	1292	90.54
Investigations	1151	80.66
Vascular disorders	1108	77.65
Respiratory, thoracic and mediastinal disorders	1060	74.28
Immune system disorders	1024	71.76
Psychiatric disorders	1016	71.20
Infections and infestations	1006	70.50
Musculoskeletal and connective tissue disorders	997	69.87
Metabolism and nutrition disorders	996	69.80
Cardiac disorders	988	69.24
Injury, poisoning and procedural complications	946	66.29
Renal and urinary disorders	911	63.84
Blood and lymphatic system disorders	885	62.02
Eye disorders	876	61.39
Hepatobiliary disorders	743	52.07
Reproductive system and breast disorders	727	50.95
Ear and labyrinth disorders	659	46.18
Neoplasms benign, malignant and unspecified (incl cysts and polyps)	376	26.35
Endocrine disorders	323	22.63
Congenital, familial and genetic disorders	253	17.73
Social circumstances	251	17.59
Surgical and medical procedures	213	14.93
Pregnancy, puerperium and perinatal conditions	125	8.76
Product issues	22	1.54

**Table 6:** Snippet of the SIDER dataset.

SMILES	RSBD	ELD	ED	BLSD
CC(=O)[O-]	0	0	0	0
CCC1=NC=CC(=C1)C(=S)N	1	0	1	1
CC(CC1=CC=CC=C1)NC	1	0	0	0

**Table 7:** Comparison of molecular fingerprints.

Fingerprint Type	Base Logic	Key Advantage	Best Use Case
RDKit Fingerprint	Path-based (Topological)	Captures linear/branched atom paths	Substructure encoding in fixed vectors
Layered Fingerprint	Hierarchical Path-based	Encodes structural complexity in layers	Bioactivity and model discrimination
MACCS Keys	Pre-defined Structural Keys	High interpretability (bits have known meanings)	Fast similarity searching and clustering
Avalon	Substructure Keys/Screening	Rapid matching across diverse patterns	Efficient screening in large databases

**Table 8:** Comparison of molecular descriptors.

Descriptor	Measured Property	Biological Significance	Key Impact
NumHDonors	H-bond donor count	Protein-ligand binding specificity	Membrane permeability and solubility
NumHAcceptors	H-bond acceptor count	Interaction with water and targets	Bioavailability and binding affinity
TPSA	Polar surface area (2D)	Molecular polarity and transport	Blood–Brain Barrier (BBB) penetration
MolLogP	Octanol-water partition	Lipophilicity/Hydrophobicity	Absorption, distribution and toxicity
NumRotatableBonds	Molecular flexibility	Conformational entropy	Target binding fit and pharmacokinetics

## Data Availability

Dataset available on request from the authors.

## Supplementary Materials

The following supporting information can be downloaded at: https://www.mdpi.com/article/10.3390/ph19101566/s1, Table S1: Detailed individual RF, SVM, and XGB results; Table S2: Per-iteration performance of the baseline hybrid model across 25 repeated cross-validation iterations; Table S3: Per-iteration performance of the Balanced Thresholds strategy across 25 repeated cross-validation iterations; Table S4: Per-iteration performance of the Weighted Predictions strategy across 25 repeated cross-validation iterations; Figure S1: Critical Difference diagram for Recall across the evaluated hybrid decision strategies. Average ranks were calculated for the Baseline Hybrid model (BH), the Balanced Thresholds strategy (BT), and the Weighted Predictions strategy (WP) across the evaluated system organ classes and repeated cross-validation iterations. Lower average ranks indicate better recall performance; Figure S2: Critical Difference diagram for Precision across the evaluated hybrid decision strategies. Average ranks were calculated for the Baseline Hybrid model (BH), the Balanced Thresholds strategy (BT), and the Weighted Predictions strategy (WP) across the evaluated system organ classes and repeated cross-validation iterations. Lower average ranks indicate better precision performance; Figure S3: Critical Difference diagram for F1-score across the evaluated hybrid decision strategies. Average ranks were calculated for the Baseline Hybrid model (BH), the Balanced Thresholds strategy (BT), and the Weighted Predictions strategy (WP) across the evaluated system organ classes and repeated cross-validation iterations. Lower average ranks indicate better F1-score performance; Figure S4: Critical Difference diagram for AUC-PR across the evaluated hybrid decision strategies. Average ranks were calculated for the Baseline Hybrid model (BH), the Balanced Thresholds strategy (BT), and the Weighted Predictions strategy (WP) across the evaluated system organ classes and repeated cross-validation iterations. Lower average ranks indicate better area under the precision–recall curve (AUC-PR) performance.

## Abbreviations

The following abbreviations are used in this manuscript:

ADR	Adverse Drug Reaction
RF	Random Forest
XGB	Extreme Gradient Boosting
SVM	Support Vector Machine
ML	Machine Learning
SIDER	Side Effect Resource
MedDRA	Medical Dictionary for Regulatory Activities
SOC	System Organ Class
RSBD	Reproductive System and Breast Disorders
ELD	Ear and Labyrinth Disorders
ED	Eye Disorders
BLSD	Blood and Lymphatic System Disorders
NumHDonors	Number of Hydrogen Bond Donors
NumHAcceptors	Number of Hydrogen Bond Acceptors
TPSA	Topological Polar Surface Area
MolLogP	Log of Octanol/Water Partition Coefficient
NumRotatableBonds	Number of Rotatable Bonds
BBB	Blood–Brain Barrier
MW	Molecular Weight
RBF	Radial Basis Function
AUC-ROC	Area Under the Operating Characteristic Curve
AUC-PR	Area Under the Precision-Recall Curve
FPR	False Positive Rate
BLB	Blood–Labyrinth Barrier
CD	Critical Difference
BH	Baseline Hybrid
BT	Balanced Thresholds
WP	Weighted Predictions
EHR	Electronic Health Records
XAI	Explainable Artificial Intelligence
ARTEMIS	Artificial Intelligence, Data Science, and Smart Engineering
ROAR-NET	Randomized Optimization Algorithms Research Network
