# Supplementary material for: Multi-Label Classification of Adverse Drug Reactions Using a Hybrid Approach Integrating Support Vector Machine, Random Forest and Extreme Gradient Boosting
Source: Pharmaceuticals (Basel). 2026 Oct 1;19(10):1566. doi: 10.3390/ph19101566 (PMC13708647; doi:10.3390/ph19101566)
Supplement: Supplementary file 1 [file pharmaceuticals-19-01566-s001.zip › pharmaceuticals-4536230-supplementary.pdf]

Supplementary Materials

Multi-label Classification of Adverse Drug Reactions Using a Hybrid Approach Integrating Support Vector Machine, Random Forest and Extreme Gradient Boosting

Ana-Maria Cucos[1], Zsolt Gáll[2,*] and László Barna Iantovics[3]

1 Doctoral School of Letters, Humanities and Applied Sciences, George Emil Palade University of Medicine, Pharmacy, Sciences and Technology of Targu Mures, Târgu Mures, Romania; ana_mariacucos@yahoo.com

2 Department of Pharmacology and Clinical Pharmacy, Faculty of Pharmacy, George Emil Palade University of Medicine, Pharmacy, Science, and Technology of Targu Mures, Romania; zsolt.gall@umfst.ro

3 Electrical Engineering and Information Technology Department, George Emil Palade University of Medicine, Pharmacy, Sciences and Technology of Targu Mures, Târgu Mures, Romania; barna.iantovics@umfst.ro

* Correspondence: zsolt.gall@umfst.ro

Table S1: Detailed individual RF, SVM, and XGB results

Iteration	Label	Model	Recall	Precision	F1-Score	AUC-PR
1	RSBD	RF	0.73	0.67	0.70	0.75
1	ELD	RF	0.52	0.61	0.56	0.65
1	ED	RF	0.92	0.65	0.76	0.78
1	BLSD	RF	0.80	0.71	0.75	0.81
1	RSBD	SVM	0.59	0.59	0.59	0.58
1	ELD	SVM	0.01	1.00	0.01	0.57
1	ED	SVM	0.97	0.62	0.76	0.77
1	BLSD	SVM	0.98	0.61	0.75	0.76
1	RSBD	XGB	0.71	0.68	0.70	0.76
1	ELD	XGB	0.49	0.62	0.55	0.70
1	ED	XGB	0.85	0.67	0.75	0.76
1	BLSD	XGB	0.83	0.67	0.74	0.80
2	RSBD	RF	0.77	0.68	0.72	0.77
2	ELD	RF	0.51	0.66	0.58	0.67
2	ED	RF	0.92	0.67	0.78	0.80
2	BLSD	RF	0.84	0.72	0.78	0.87
2	RSBD	SVM	0.69	0.64	0.67	0.61
2	ELD	SVM	0.39	0.64	0.48	0.62
2	ED	SVM	0.99	0.61	0.76	0.77
2	BLSD	SVM	0.99	0.63	0.77	0.83
2	RSBD	XGB	0.68	0.68	0.68	0.79
2	ELD	XGB	0.49	0.68	0.57	0.71
2	ED	XGB	0.93	0.66	0.77	0.80
2	BLSD	XGB	0.89	0.74	0.81	0.85
3	RSBD	RF	0.70	0.65	0.67	0.76

3	ELD	RF	0.40	0.66	0.50	0.68
3	ED	RF	0.86	0.69	0.77	0.78
3	BLSD	RF	0.77	0.69	0.73	0.78
3	RSBD	SVM	0.62	0.65	0.63	0.66
3	ELD	SVM	0.33	0.65	0.44	0.64
3	ED	SVM	0.97	0.66	0.78	0.74
3	BLSD	SVM	0.99	0.59	0.74	0.72
3	RSBD	XGB	0.73	0.68	0.70	0.76
3	ELD	XGB	0.43	0.69	0.53	0.71
3	ED	XGB	0.89	0.69	0.78	0.79
3	BLSD	XGB	0.86	0.66	0.75	0.80
4	RSBD	RF	0.75	0.67	0.71	0.75
4	ELD	RF	0.58	0.58	0.58	0.61
4	ED	RF	0.88	0.65	0.75	0.71
4	BLSD	RF	0.78	0.75	0.76	0.83
4	RSBD	SVM	0.76	0.59	0.66	0.65
4	ELD	SVM	0.02	1.00	0.03	0.49
4	ED	SVM	0.98	0.61	0.75	0.69
4	BLSD	SVM	1.00	0.67	0.81	0.77
4	RSBD	XGB	0.74	0.68	0.71	0.77
4	ELD	XGB	0.51	0.54	0.52	0.62
4	ED	XGB	0.92	0.66	0.77	0.75
4	BLSD	XGB	0.87	0.73	0.80	0.83
5	RSBD	RF	0.74	0.63	0.68	0.75
5	ELD	RF	0.56	0.58	0.57	0.64
5	ED	RF	0.96	0.65	0.77	0.81
5	BLSD	RF	0.87	0.69	0.77	0.83
5	RSBD	SVM	0.68	0.61	0.64	0.60
5	ELD	SVM	0.00	0.00	0.00	0.51
5	ED	SVM	0.99	0.60	0.75	0.69
5	BLSD	SVM	0.97	0.61	0.75	0.80
5	RSBD	XGB	0.72	0.67	0.69	0.77
5	ELD	XGB	0.55	0.59	0.57	0.64
5	ED	XGB	0.98	0.65	0.78	0.81
5	BLSD	XGB	0.92	0.69	0.79	0.85
6	RSBD	RF	0.73	0.64	0.68	0.76
6	ELD	RF	0.47	0.55	0.51	0.61
6	ED	RF	0.90	0.70	0.79	0.80
6	BLSD	RF	0.83	0.70	0.76	0.85
6	RSBD	SVM	0.64	0.57	0.60	0.63
6	ELD	SVM	0.32	0.55	0.41	0.55
6	ED	SVM	0.98	0.65	0.78	0.79
6	BLSD	SVM	0.99	0.62	0.76	0.83
6	RSBD	XGB	0.80	0.64	0.71	0.74
6	ELD	XGB	0.48	0.58	0.53	0.61
6	ED	XGB	0.87	0.68	0.76	0.80

6	BLSD	XGB	0.91	0.67	0.77	0.84
7	RSBD	RF	0.76	0.72	0.74	0.83
7	ELD	RF	0.50	0.69	0.58	0.70
7	ED	RF	0.94	0.63	0.75	0.75
7	BLSD	RF	0.78	0.77	0.77	0.86
7	RSBD	SVM	0.69	0.71	0.70	0.70
7	ELD	SVM	0.25	0.61	0.35	0.65
7	ED	SVM	0.95	0.61	0.74	0.73
7	BLSD	SVM	0.96	0.69	0.81	0.83
7	RSBD	XGB	0.74	0.76	0.75	0.82
7	ELD	XGB	0.49	0.70	0.58	0.71
7	ED	XGB	0.95	0.64	0.77	0.78
7	BLSD	XGB	0.84	0.76	0.80	0.88
8	RSBD	RF	0.68	0.66	0.67	0.71
8	ELD	RF	0.53	0.61	0.57	0.68
8	ED	RF	0.91	0.67	0.77	0.75
8	BLSD	RF	0.82	0.70	0.75	0.82
8	RSBD	SVM	0.64	0.60	0.62	0.59
8	ELD	SVM	0.33	0.65	0.44	0.60
8	ED	SVM	0.99	0.62	0.76	0.71
8	BLSD	SVM	1.00	0.61	0.76	0.80
8	RSBD	XGB	0.66	0.68	0.67	0.75
8	ELD	XGB	0.54	0.65	0.59	0.68
8	ED	XGB	0.86	0.66	0.75	0.75
8	BLSD	XGB	0.84	0.69	0.76	0.80
9	RSBD	RF	0.79	0.61	0.69	0.75
9	ELD	RF	0.50	0.53	0.51	0.57
9	ED	RF	0.93	0.59	0.72	0.67
9	BLSD	RF	0.86	0.67	0.75	0.79
9	RSBD	SVM	0.70	0.58	0.64	0.61
9	ELD	SVM	0.06	0.28	0.09	0.45
9	ED	SVM	0.99	0.57	0.72	0.64
9	BLSD	SVM	0.99	0.59	0.74	0.71
9	RSBD	XGB	0.74	0.63	0.68	0.74
9	ELD	XGB	0.50	0.60	0.55	0.68
9	ED	XGB	0.93	0.60	0.73	0.70
9	BLSD	XGB	0.89	0.66	0.75	0.80
10	RSBD	RF	0.71	0.65	0.68	0.72
10	ELD	RF	0.47	0.60	0.53	0.63
10	ED	RF	0.88	0.75	0.81	0.80
10	BLSD	RF	0.78	0.73	0.75	0.83
10	RSBD	SVM	0.68	0.56	0.62	0.57
10	ELD	SVM	0.30	0.58	0.39	0.58
10	ED	SVM	0.95	0.66	0.78	0.75
10	BLSD	SVM	0.99	0.61	0.76	0.83
10	RSBD	XGB	0.64	0.62	0.63	0.71

10	ELD	XGB	0.43	0.60	0.50	0.61
10	ED	XGB	0.91	0.74	0.82	0.81
10	BLSD	XGB	0.84	0.69	0.76	0.83
11	RSBD	RF	0.69	0.68	0.68	0.76
11	ELD	RF	0.41	0.71	0.52	0.73
11	ED	RF	0.91	0.74	0.81	0.84
11	BLSD	RF	0.78	0.75	0.77	0.83
11	RSBD	SVM	0.71	0.62	0.66	0.68
11	ELD	SVM	0.22	0.73	0.34	0.70
11	ED	SVM	0.97	0.68	0.80	0.79
11	BLSD	SVM	0.98	0.64	0.77	0.81
11	RSBD	XGB	0.69	0.70	0.70	0.77
11	ELD	XGB	0.45	0.73	0.56	0.72
11	ED	XGB	0.85	0.74	0.80	0.81
11	BLSD	XGB	0.80	0.72	0.76	0.83
12	RSBD	RF	0.73	0.70	0.71	0.77
12	ELD	RF	0.54	0.62	0.58	0.66
12	ED	RF	0.88	0.66	0.75	0.77
12	BLSD	RF	0.80	0.65	0.72	0.84
12	RSBD	SVM	0.58	0.72	0.64	0.68
12	ELD	SVM	0.24	0.63	0.35	0.60
12	ED	SVM	0.97	0.64	0.77	0.75
12	BLSD	SVM	0.99	0.59	0.74	0.77
12	RSBD	XGB	0.65	0.70	0.68	0.75
12	ELD	XGB	0.45	0.58	0.51	0.60
12	ED	XGB	0.88	0.65	0.75	0.77
12	BLSD	XGB	0.87	0.67	0.76	0.83
13	RSBD	RF	0.76	0.60	0.67	0.74
13	ELD	RF	0.52	0.54	0.53	0.60
13	ED	RF	0.91	0.64	0.75	0.74
13	BLSD	RF	0.83	0.73	0.78	0.84
13	RSBD	SVM	0.68	0.59	0.63	0.59
13	ELD	SVM	0.41	0.47	0.44	0.52
13	ED	SVM	0.98	0.61	0.75	0.73
13	BLSD	SVM	0.99	0.65	0.78	0.82
13	RSBD	XGB	0.73	0.64	0.68	0.74
13	ELD	XGB	0.46	0.62	0.53	0.66
13	ED	XGB	0.89	0.67	0.77	0.75
13	BLSD	XGB	0.88	0.73	0.80	0.84
14	RSBD	RF	0.71	0.68	0.70	0.77
14	ELD	RF	0.56	0.60	0.58	0.64
14	ED	RF	0.89	0.65	0.75	0.76
14	BLSD	RF	0.80	0.74	0.77	0.85
14	RSBD	SVM	0.65	0.64	0.64	0.59
14	ELD	SVM	0.31	0.54	0.40	0.53
14	ED	SVM	0.97	0.63	0.76	0.76

14	BLSD	SVM	0.96	0.64	0.77	0.80
14	RSBD	XGB	0.67	0.72	0.69	0.79
14	ELD	XGB	0.56	0.62	0.59	0.64
14	ED	XGB	0.89	0.66	0.76	0.76
14	BLSD	XGB	0.87	0.70	0.78	0.84
15	RSBD	RF	0.77	0.66	0.71	0.78
15	ELD	RF	0.49	0.55	0.52	0.60
15	ED	RF	0.94	0.61	0.74	0.74
15	BLSD	RF	0.82	0.70	0.76	0.79
15	RSBD	SVM	0.67	0.56	0.61	0.60
15	ELD	SVM	0.00	0.00	0.00	0.47
15	ED	SVM	0.99	0.57	0.72	0.69
15	BLSD	SVM	0.99	0.61	0.75	0.75
15	RSBD	XGB	0.77	0.69	0.73	0.79
15	ELD	XGB	0.58	0.64	0.61	0.64
15	ED	XGB	0.92	0.62	0.74	0.74
15	BLSD	XGB	0.89	0.69	0.78	0.79
16	RSBD	RF	0.76	0.69	0.72	0.79
16	ELD	RF	0.53	0.63	0.58	0.63
16	ED	RF	0.89	0.68	0.77	0.78
16	BLSD	RF	0.84	0.70	0.76	0.81
16	RSBD	SVM	0.61	0.65	0.63	0.64
16	ELD	SVM	0.50	0.57	0.54	0.58
16	ED	SVM	0.96	0.64	0.77	0.77
16	BLSD	SVM	0.99	0.60	0.75	0.76
16	RSBD	XGB	0.73	0.74	0.73	0.82
16	ELD	XGB	0.56	0.65	0.60	0.66
16	ED	XGB	0.91	0.71	0.80	0.78
16	BLSD	XGB	0.91	0.69	0.78	0.80
17	RSBD	RF	0.72	0.65	0.69	0.74
17	ELD	RF	0.48	0.61	0.54	0.62
17	ED	RF	0.88	0.65	0.75	0.74
17	BLSD	RF	0.80	0.71	0.75	0.83
17	RSBD	SVM	0.69	0.61	0.65	0.62
17	ELD	SVM	0.22	0.62	0.32	0.58
17	ED	SVM	0.99	0.60	0.75	0.74
17	BLSD	SVM	0.99	0.64	0.77	0.78
17	RSBD	XGB	0.67	0.65	0.66	0.73
17	ELD	XGB	0.49	0.62	0.55	0.67
17	ED	XGB	0.89	0.65	0.75	0.79
17	BLSD	XGB	0.91	0.73	0.81	0.85
18	RSBD	RF	0.69	0.66	0.68	0.75
18	ELD	RF	0.49	0.65	0.56	0.64
18	ED	RF	0.94	0.71	0.81	0.79
18	BLSD	RF	0.84	0.74	0.78	0.85
18	RSBD	SVM	0.65	0.59	0.62	0.60

18	ELD	SVM	0.15	0.73	0.25	0.61
18	ED	SVM	0.96	0.66	0.78	0.75
18	BLSD	SVM	0.98	0.64	0.77	0.83
18	RSBD	XGB	0.69	0.66	0.68	0.74
18	ELD	XGB	0.47	0.66	0.55	0.66
18	ED	XGB	0.91	0.70	0.79	0.81
18	BLSD	XGB	0.86	0.71	0.77	0.84
19	RSBD	RF	0.79	0.67	0.72	0.82
19	ELD	RF	0.56	0.55	0.55	0.66
19	ED	RF	0.91	0.64	0.75	0.75
19	BLSD	RF	0.84	0.68	0.75	0.81
19	RSBD	SVM	0.72	0.63	0.67	0.69
19	ELD	SVM	0.16	0.72	0.27	0.60
19	ED	SVM	0.97	0.61	0.75	0.72
19	BLSD	SVM	0.99	0.61	0.75	0.79
19	RSBD	XGB	0.75	0.72	0.74	0.84
19	ELD	XGB	0.60	0.61	0.61	0.67
19	ED	XGB	0.92	0.66	0.77	0.77
19	BLSD	XGB	0.90	0.68	0.78	0.83
20	RSBD	RF	0.73	0.65	0.69	0.75
20	ELD	RF	0.50	0.60	0.55	0.67
20	ED	RF	0.95	0.63	0.76	0.74
20	BLSD	RF	0.80	0.74	0.77	0.85
20	RSBD	SVM	0.64	0.60	0.62	0.59
20	ELD	SVM	0.35	0.59	0.44	0.55
20	ED	SVM	0.99	0.59	0.74	0.72
20	BLSD	SVM	0.99	0.65	0.78	0.83
20	RSBD	XGB	0.73	0.66	0.69	0.79
20	ELD	XGB	0.51	0.62	0.56	0.67
20	ED	XGB	0.93	0.63	0.75	0.75
20	BLSD	XGB	0.86	0.70	0.77	0.83
21	RSBD	RF	0.71	0.66	0.68	0.76
21	ELD	RF	0.48	0.55	0.51	0.63
21	ED	RF	0.91	0.66	0.77	0.80
21	BLSD	RF	0.80	0.74	0.77	0.83
21	RSBD	SVM	0.66	0.62	0.64	0.67
21	ELD	SVM	0.05	0.50	0.10	0.50
21	ED	SVM	0.99	0.62	0.77	0.76
21	BLSD	SVM	0.99	0.65	0.78	0.82
21	RSBD	XGB	0.69	0.72	0.71	0.77
21	ELD	XGB	0.56	0.61	0.58	0.66
21	ED	XGB	0.93	0.69	0.79	0.79
21	BLSD	XGB	0.86	0.73	0.79	0.85
22	RSBD	RF	0.66	0.69	0.67	0.76
22	ELD	RF	0.47	0.56	0.51	0.62
22	ED	RF	0.90	0.65	0.76	0.78

22	BLSD	RF	0.77	0.68	0.72	0.80
22	RSBD	SVM	0.63	0.70	0.66	0.68
22	ELD	SVM	0.22	0.73	0.33	0.60
22	ED	SVM	0.98	0.63	0.77	0.75
22	BLSD	SVM	0.98	0.64	0.78	0.75
22	RSBD	XGB	0.69	0.69	0.69	0.79
22	ELD	XGB	0.50	0.60	0.54	0.64
22	ED	XGB	0.92	0.65	0.76	0.79
22	BLSD	XGB	0.85	0.69	0.76	0.82
23	RSBD	RF	0.74	0.61	0.67	0.72
23	ELD	RF	0.51	0.56	0.53	0.62
23	ED	RF	0.87	0.66	0.75	0.71
23	BLSD	RF	0.84	0.71	0.77	0.84
23	RSBD	SVM	0.63	0.60	0.62	0.61
23	ELD	SVM	0.44	0.56	0.49	0.59
23	ED	SVM	0.97	0.63	0.76	0.69
23	BLSD	SVM	0.98	0.64	0.78	0.80
23	RSBD	XGB	0.71	0.66	0.69	0.74
23	ELD	XGB	0.52	0.60	0.56	0.61
23	ED	XGB	0.83	0.67	0.74	0.74
23	BLSD	XGB	0.90	0.73	0.80	0.83
24	RSBD	RF	0.75	0.70	0.72	0.82
24	ELD	RF	0.52	0.72	0.61	0.72
24	ED	RF	0.92	0.67	0.78	0.78
24	BLSD	RF	0.84	0.69	0.76	0.83
24	RSBD	SVM	0.71	0.63	0.67	0.61
24	ELD	SVM	0.08	0.86	0.15	0.67
24	ED	SVM	0.98	0.64	0.77	0.75
24	BLSD	SVM	0.99	0.59	0.74	0.80
24	RSBD	XGB	0.73	0.69	0.71	0.80
24	ELD	XGB	0.49	0.72	0.58	0.70
24	ED	XGB	0.94	0.67	0.78	0.79
24	BLSD	XGB	0.88	0.68	0.77	0.81
25	RSBD	RF	0.76	0.66	0.71	0.74
25	ELD	RF	0.45	0.55	0.50	0.60
25	ED	RF	0.89	0.64	0.74	0.72
25	BLSD	RF	0.82	0.73	0.78	0.85
25	RSBD	SVM	0.71	0.57	0.63	0.58
25	ELD	SVM	0.07	0.36	0.11	0.47
25	ED	SVM	0.97	0.60	0.74	0.71
25	BLSD	SVM	0.99	0.62	0.77	0.81
25	RSBD	XGB	0.76	0.68	0.72	0.76
25	ELD	XGB	0.43	0.57	0.49	0.64
25	ED	XGB	0.92	0.66	0.77	0.74
25	BLSD	XGB	0.92	0.72	0.81	0.85

Table S2: Per-iteration performance of the baseline hybrid model across 25 repeated cross-validation iterations.

Iteration	Label	Recall	Precision	F1-score	AUC-PR
1	RSBD	0.71	0.66	0.68	0.73
1	ELD	0.41	0.65	0.50	0.67
1	ED	0.94	0.65	0.77	0.79
1	BLSD	0.91	0.67	0.77	0.81
2	RSBD	0.76	0.69	0.72	0.75
2	ELD	0.47	0.69	0.56	0.68
2	ED	0.98	0.63	0.77	0.81
2	BLSD	0.92	0.70	0.80	0.87
3	RSBD	0.70	0.67	0.69	0.79
3	ELD	0.38	0.69	0.49	0.70
3	ED	0.91	0.69	0.78	0.79
3	BLSD	0.90	0.65	0.75	0.79
4	RSBD	0.74	0.67	0.71	0.78
4	ELD	0.51	0.60	0.55	0.62
4	ED	0.92	0.63	0.75	0.74
4	BLSD	0.91	0.72	0.80	0.84
5	RSBD	0.70	0.64	0.67	0.74
5	ELD	0.52	0.65	0.57	0.65
5	ED	0.98	0.63	0.77	0.81
5	BLSD	0.94	0.67	0.78	0.84
6	RSBD	0.76	0.65	0.70	0.75
6	ELD	0.48	0.60	0.53	0.62
6	ED	0.94	0.68	0.79	0.81
6	BLSD	0.94	0.65	0.77	0.85
7	RSBD	0.74	0.74	0.74	0.82
7	ELD	0.45	0.72	0.55	0.71
7	ED	0.93	0.63	0.75	0.77
7	BLSD	0.87	0.74	0.80	0.87
8	RSBD	0.61	0.67	0.64	0.71
8	ELD	0.48	0.63	0.54	0.68
8	ED	0.95	0.65	0.77	0.76
8	BLSD	0.92	0.66	0.77	0.82
9	RSBD	0.79	0.61	0.69	0.75
9	ELD	0.41	0.61	0.49	0.61
9	ED	0.96	0.59	0.73	0.69
9	BLSD	0.93	0.64	0.76	0.79
10	RSBD	0.70	0.64	0.67	0.69
10	ELD	0.42	0.67	0.52	0.63
10	ED	0.95	0.72	0.82	0.81
10	BLSD	0.93	0.69	0.79	0.83

11	RSBD	0.72	0.70	0.71	0.78
11	ELD	0.37	0.73	0.49	0.74
11	ED	0.91	0.72	0.80	0.84
11	BLSD	0.89	0.72	0.80	0.83
12	RSBD	0.65	0.70	0.67	0.75
12	ELD	0.45	0.62	0.52	0.65
12	ED	0.93	0.64	0.76	0.78
12	BLSD	0.89	0.61	0.73	0.84
13	RSBD	0.74	0.63	0.68	0.74
13	ELD	0.45	0.57	0.50	0.61
13	ED	0.95	0.64	0.77	0.75
13	BLSD	0.92	0.71	0.80	0.85
14	RSBD	0.69	0.70	0.70	0.75
14	ELD	0.50	0.63	0.56	0.64
14	ED	0.94	0.65	0.77	0.78
14	BLSD	0.92	0.71	0.80	0.85
15	RSBD	0.78	0.67	0.72	0.80
15	ELD	0.49	0.63	0.55	0.63
15	ED	0.97	0.60	0.74	0.76
15	BLSD	0.92	0.66	0.77	0.79
16	RSBD	0.77	0.74	0.75	0.79
16	ELD	0.52	0.64	0.57	0.64
16	ED	0.92	0.67	0.77	0.79
16	BLSD	0.94	0.66	0.77	0.81
17	RSBD	0.72	0.64	0.68	0.76
17	ELD	0.43	0.64	0.51	0.64
17	ED	0.96	0.63	0.76	0.77
17	BLSD	0.93	0.69	0.79	0.84
18	RSBD	0.67	0.66	0.67	0.71
18	ELD	0.41	0.67	0.50	0.67
18	ED	0.95	0.70	0.81	0.81
18	BLSD	0.93	0.70	0.80	0.85
19	RSBD	0.79	0.69	0.74	0.83
19	ELD	0.50	0.62	0.56	0.67
19	ED	0.96	0.63	0.76	0.77
19	BLSD	0.94	0.65	0.76	0.82
20	RSBD	0.74	0.66	0.70	0.76
20	ELD	0.48	0.64	0.55	0.67
20	ED	0.97	0.61	0.75	0.76
20	BLSD	0.92	0.70	0.79	0.85
21	RSBD	0.69	0.68	0.68	0.77
21	ELD	0.43	0.58	0.49	0.64
21	ED	0.97	0.66	0.78	0.80
21	BLSD	0.90	0.69	0.78	0.85
22	RSBD	0.67	0.72	0.70	0.77
22	ELD	0.46	0.61	0.52	0.64

22	ED	0.96	0.65	0.78	0.79
22	BLSD	0.87	0.67	0.75	0.80
23	RSBD	0.72	0.64	0.68	0.74
23	ELD	0.49	0.60	0.54	0.62
23	ED	0.90	0.65	0.76	0.73
23	BLSD	0.94	0.70	0.80	0.84
24	RSBD	0.73	0.69	0.71	0.81
24	ELD	0.43	0.77	0.56	0.74
24	ED	0.95	0.66	0.78	0.80
24	BLSD	0.94	0.64	0.76	0.83
25	RSBD	0.77	0.66	0.71	0.74
25	ELD	0.41	0.58	0.48	0.61
25	ED	0.95	0.64	0.77	0.73
25	BLSD	0.96	0.70	0.81	0.86

Table S3: Per-iteration performance of the Balanced Thresholds strategy across 25 repeated cross-validation iterations.

Iteration	Label	Recall	Precision	F1-score	AUC-PR
1	RSBD	0.93	0.58	0.72	0.73
1	ELD	0.92	0.53	0.67	0.67
1	ED	1.00	0.62	0.76	0.79
1	BLSD	0.99	0.61	0.76	0.81
2	RSBD	0.95	0.57	0.71	0.75
2	ELD	0.98	0.51	0.67	0.68
2	ED	1.00	0.61	0.76	0.81
2	BLSD	1.00	0.63	0.77	0.87
3	RSBD	0.93	0.56	0.70	0.79
3	ELD	0.91	0.53	0.67	0.70
3	ED	1.00	0.64	0.78	0.79
3	BLSD	1.00	0.59	0.74	0.79
4	RSBD	0.94	0.52	0.67	0.78
4	ELD	0.93	0.43	0.59	0.62
4	ED	1.00	0.61	0.76	0.74
4	BLSD	1.00	0.67	0.80	0.84
5	RSBD	0.95	0.57	0.71	0.74
5	ELD	1.00	0.45	0.62	0.65
5	ED	1.00	0.59	0.74	0.81
5	BLSD	1.00	0.61	0.76	0.84
6	RSBD	0.96	0.54	0.69	0.75
6	ELD	0.89	0.47	0.62	0.62
6	ED	0.99	0.63	0.77	0.81
6	BLSD	1.00	0.61	0.76	0.85
7	RSBD	0.95	0.65	0.77	0.82
7	ELD	0.96	0.53	0.69	0.71
7	ED	1.00	0.60	0.75	0.77

7	BLSD	0.99	0.69	0.81	0.87
8	RSBD	0.95	0.59	0.72	0.71
8	ELD	0.95	0.47	0.63	0.68
8	ED	1.00	0.61	0.76	0.76
8	BLSD	1.00	0.61	0.76	0.82
9	RSBD	0.96	0.51	0.66	0.75
9	ELD	0.97	0.44	0.60	0.61
9	ED	1.00	0.56	0.72	0.69
9	BLSD	0.99	0.59	0.74	0.79
10	RSBD	0.94	0.55	0.69	0.69
10	ELD	0.90	0.52	0.66	0.63
10	ED	1.00	0.66	0.80	0.81
10	BLSD	0.99	0.61	0.76	0.83
11	RSBD	0.99	0.57	0.72	0.78
11	ELD	0.94	0.59	0.72	0.74
11	ED	1.00	0.67	0.81	0.84
11	BLSD	1.00	0.63	0.77	0.83
12	RSBD	0.92	0.62	0.74	0.75
12	ELD	0.95	0.48	0.63	0.65
12	ED	1.00	0.62	0.76	0.78
12	BLSD	1.00	0.58	0.73	0.84
13	RSBD	0.92	0.52	0.66	0.74
13	ELD	0.89	0.46	0.61	0.61
13	ED	1.00	0.60	0.75	0.75
13	BLSD	1.00	0.65	0.79	0.85
14	RSBD	0.94	0.57	0.71	0.75
14	ELD	0.93	0.49	0.64	0.64
14	ED	1.00	0.61	0.76	0.78
14	BLSD	0.99	0.64	0.78	0.85
15	RSBD	0.97	0.53	0.69	0.80
15	ELD	0.99	0.41	0.58	0.63
15	ED	1.00	0.56	0.72	0.76
15	BLSD	1.00	0.60	0.75	0.79
16	RSBD	0.96	0.60	0.74	0.79
16	ELD	0.95	0.50	0.65	0.64
16	ED	1.00	0.64	0.78	0.79
16	BLSD	0.99	0.60	0.75	0.81
17	RSBD	0.94	0.52	0.67	0.76
17	ELD	0.98	0.47	0.64	0.64
17	ED	1.00	0.59	0.74	0.77
17	BLSD	1.00	0.63	0.78	0.84
18	RSBD	0.93	0.57	0.71	0.71
18	ELD	0.85	0.52	0.65	0.67
18	ED	1.00	0.65	0.79	0.81
18	BLSD	1.00	0.63	0.77	0.85
19	RSBD	0.94	0.55	0.70	0.83

19	ELD	0.95	0.48	0.64	0.67
19	ED	1.00	0.60	0.75	0.77
19	BLSD	1.00	0.60	0.75	0.82
20	RSBD	0.94	0.56	0.70	0.76
20	ELD	0.94	0.47	0.63	0.67
20	ED	1.00	0.58	0.74	0.76
20	BLSD	1.00	0.64	0.78	0.85
21	RSBD	0.91	0.58	0.71	0.77
21	ELD	0.96	0.47	0.63	0.64
21	ED	1.00	0.61	0.76	0.80
21	BLSD	1.00	0.64	0.78	0.85
22	RSBD	0.91	0.61	0.73	0.77
22	ELD	0.96	0.50	0.66	0.64
22	ED	1.00	0.62	0.77	0.79
22	BLSD	0.99	0.64	0.78	0.80
23	RSBD	0.93	0.54	0.68	0.74
23	ELD	0.92	0.49	0.64	0.62
23	ED	1.00	0.61	0.76	0.73
23	BLSD	1.00	0.63	0.77	0.84
24	RSBD	0.99	0.55	0.71	0.81
24	ELD	0.95	0.54	0.69	0.74
24	ED	1.00	0.63	0.77	0.80
24	BLSD	1.00	0.59	0.74	0.83
25	RSBD	0.96	0.53	0.68	0.74
25	ELD	1.00	0.43	0.60	0.61
25	ED	1.00	0.59	0.74	0.73
25	BLSD	1.00	0.62	0.76	0.86

Table S4: Per-iteration performance of the Weighted Predictions strategy across 25 repeated cross-validation iterations.

Iteration	Label	Recall	Precision	F1-score	AUC-PR
1	RSBD	0.99	0.54	0.70	0.66
1	ELD	1.00	0.49	0.66	0.59
1	ED	1.00	0.62	0.76	0.72
1	BLSD	0.99	0.61	0.76	0.72
2	RSBD	0.99	0.55	0.71	0.67
2	ELD	1.00	0.48	0.65	0.63
2	ED	1.00	0.61	0.76	0.78
2	BLSD	1.00	0.63	0.77	0.79
3	RSBD	0.96	0.54	0.69	0.66
3	ELD	1.00	0.48	0.65	0.61
3	ED	1.00	0.64	0.78	0.74
3	BLSD	0.99	0.59	0.74	0.76
4	RSBD	0.99	0.49	0.65	0.67

4	ELD	1.00	0.42	0.59	0.55
4	ED	1.00	0.61	0.76	0.69
4	BLSD	1.00	0.67	0.81	0.77
5	RSBD	0.98	0.55	0.71	0.64
5	ELD	1.00	0.44	0.61	0.57
5	ED	1.00	0.59	0.74	0.74
5	BLSD	1.00	0.61	0.76	0.76
6	RSBD	0.98	0.50	0.66	0.63
6	ELD	1.00	0.45	0.62	0.55
6	ED	0.99	0.63	0.77	0.76
6	BLSD	1.00	0.61	0.76	0.76
7	RSBD	0.99	0.61	0.75	0.72
7	ELD	1.00	0.49	0.66	0.64
7	ED	1.00	0.60	0.75	0.71
7	BLSD	0.99	0.69	0.81	0.80
8	RSBD	0.96	0.56	0.70	0.64
8	ELD	1.00	0.45	0.62	0.55
8	ED	1.00	0.61	0.76	0.70
8	BLSD	1.00	0.61	0.76	0.76
9	RSBD	0.99	0.50	0.66	0.64
9	ELD	1.00	0.44	0.61	0.54
9	ED	1.00	0.56	0.72	0.63
9	BLSD	0.99	0.59	0.74	0.72
10	RSBD	0.98	0.52	0.68	0.63
10	ELD	1.00	0.48	0.65	0.58
10	ED	1.00	0.66	0.80	0.77
10	BLSD	0.99	0.62	0.76	0.77
11	RSBD	1.00	0.52	0.68	0.70
11	ELD	1.00	0.52	0.68	0.69
11	ED	1.00	0.67	0.81	0.81
11	BLSD	0.99	0.63	0.77	0.79
12	RSBD	0.95	0.57	0.71	0.69
12	ELD	1.00	0.45	0.62	0.55
12	ED	1.00	0.62	0.76	0.72
12	BLSD	0.99	0.58	0.73	0.73
13	RSBD	0.97	0.52	0.68	0.62
13	ELD	1.00	0.46	0.63	0.54
13	ED	1.00	0.60	0.75	0.70
13	BLSD	1.00	0.65	0.79	0.77
14	RSBD	0.98	0.55	0.70	0.67
14	ELD	1.00	0.47	0.64	0.58
14	ED	1.00	0.61	0.76	0.70
14	BLSD	0.99	0.64	0.78	0.77
15	RSBD	1.00	0.49	0.66	0.68
15	ELD	1.00	0.41	0.58	0.57
15	ED	1.00	0.56	0.72	0.66

15	BLSD	1.00	0.60	0.75	0.75
16	RSBD	0.99	0.56	0.72	0.73
16	ELD	1.00	0.46	0.63	0.58
16	ED	1.00	0.64	0.78	0.73
16	BLSD	0.99	0.60	0.75	0.73
17	RSBD	0.96	0.51	0.66	0.64
17	ELD	1.00	0.45	0.62	0.59
17	ED	1.00	0.59	0.74	0.71
17	BLSD	0.99	0.63	0.77	0.76
18	RSBD	0.97	0.55	0.70	0.64
18	ELD	1.00	0.50	0.67	0.62
18	ED	1.00	0.65	0.79	0.76
18	BLSD	1.00	0.63	0.78	0.77
19	RSBD	0.99	0.53	0.69	0.69
19	ELD	1.00	0.45	0.62	0.55
19	ED	1.00	0.60	0.75	0.70
19	BLSD	1.00	0.61	0.75	0.74
20	RSBD	0.99	0.54	0.69	0.66
20	ELD	1.00	0.45	0.62	0.59
20	ED	1.00	0.58	0.74	0.71
20	BLSD	1.00	0.64	0.78	0.79
21	RSBD	0.98	0.57	0.72	0.67
21	ELD	1.00	0.46	0.63	0.56
21	ED	1.00	0.61	0.76	0.74
21	BLSD	1.00	0.64	0.78	0.79
22	RSBD	0.96	0.58	0.72	0.69
22	ELD	1.00	0.47	0.64	0.55
22	ED	1.00	0.62	0.77	0.72
22	BLSD	0.99	0.64	0.78	0.74
23	RSBD	0.97	0.52	0.68	0.63
23	ELD	1.00	0.46	0.63	0.56
23	ED	1.00	0.61	0.76	0.71
23	BLSD	0.99	0.63	0.77	0.75
24	RSBD	1.00	0.52	0.69	0.69
24	ELD	1.00	0.51	0.67	0.65
24	ED	1.00	0.63	0.77	0.73
24	BLSD	1.00	0.59	0.74	0.75
25	RSBD	1.00	0.49	0.66	0.67
25	ELD	1.00	0.42	0.59	0.53
25	ED	1.00	0.59	0.74	0.70
25	BLSD	1.00	0.62	0.76	0.79

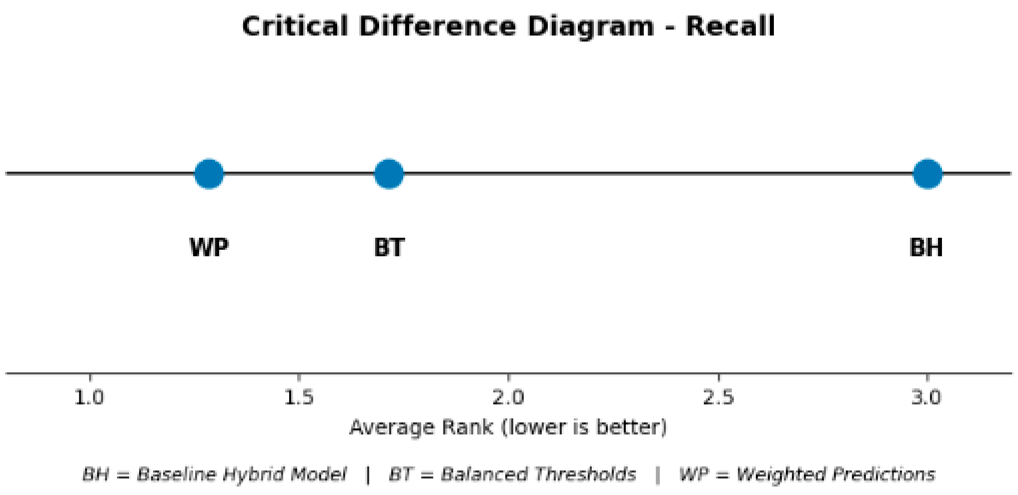

Figure S1. Critical Difference diagram for Recall across the evaluated hybrid decision strategies. Average ranks were calculated for the Baseline Hybrid model (BH), the Balanced Thresholds strategy (BT), and the Weighted Predictions strategy (WP) across the evaluated system organ classes and repeated cross-validation iterations. Lower average ranks indicate better recall performance.

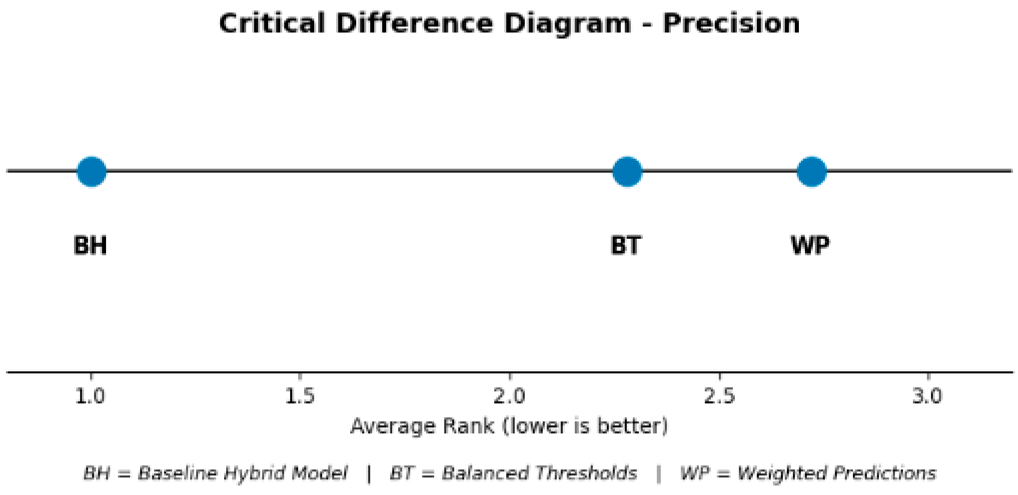

Figure S2. Critical Difference diagram for Precision across the evaluated hybrid decision strategies. Average ranks were calculated for the Baseline Hybrid model (BH), the Balanced Thresholds strategy (BT), and the Weighted Predictions strategy (WP) across the evaluated system organ classes and repeated cross-validation iterations. Lower average ranks indicate better precision performance.

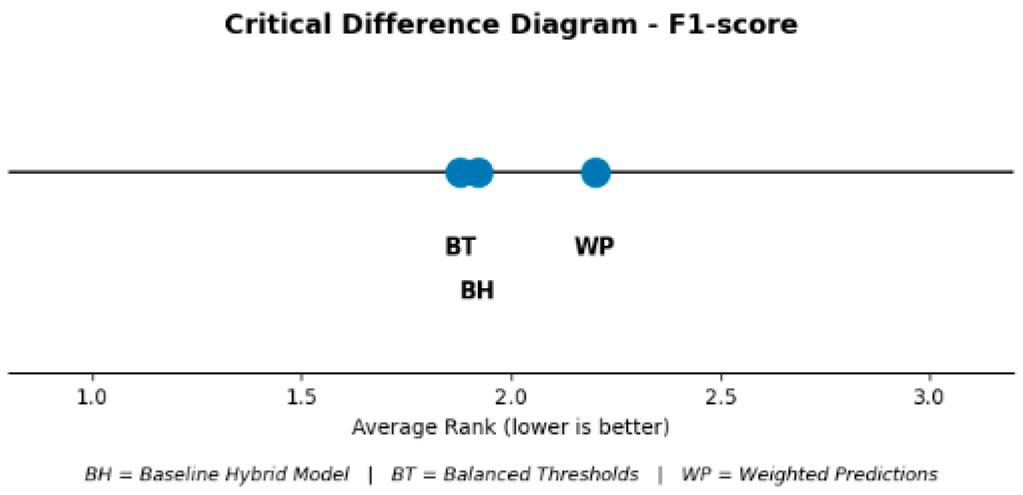

Figure S3. Critical Difference diagram for F1-score across the evaluated hybrid decision strategies. Average ranks were calculated for the Baseline Hybrid model (BH), the Balanced Thresholds strategy (BT), and the Weighted Predictions strategy (WP) across the evaluated system organ classes and repeated cross-validation iterations. Lower average ranks indicate better F1-score performance.

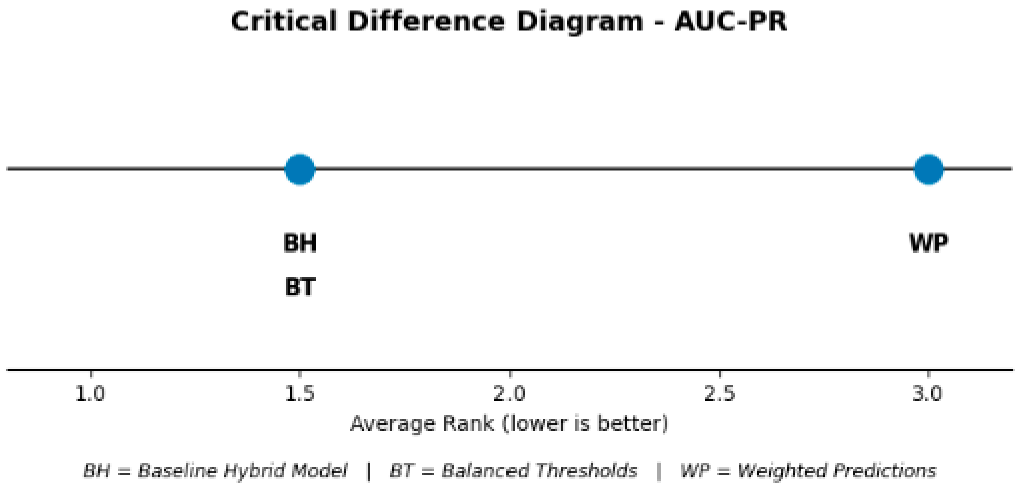

Figure S4. Critical Difference diagram for AUC-PR across the evaluated hybrid decision strategies. Average ranks were calculated for the Baseline Hybrid model (BH), the Balanced Thresholds strategy (BT), and the Weighted Predictions strategy (WP) across the evaluated system organ classes and repeated cross-validation iterations. Lower average ranks indicate better area under the precision–recall curve (AUC-PR) performance.
